# Gene–Environment Correlation over Time: A Longitudinal Analysis of Polygenic Risk Scores for Schizophrenia and Major Depression in Three British Cohorts Studies

**DOI:** 10.3390/genes13071136

**Published:** 2022-06-24

**Authors:** Sandra Machlitt-Northen, Robert Keers, Patricia B. Munroe, David M. Howard, Michael Pluess

**Affiliations:** 1 Department of Biological and Experimental Psychology, Queen Mary University of London, London E1 4NS, UK; s.machlitt-northen@qmul.ac.uk; 2 Department of Clinical Pharmacology, William Harvey Research Institute, Queen Mary University of London, Charterhouse Square, London EC1M 6BQ, UK; p.b.munroe@qmul.ac.uk; 3 Social, Genetic and Developmental Psychiatry Centre, King’s College London, London SE5 8AF, UK; david.howard@kcl.ac.uk; 4 Division of Psychiatry, University of Edinburgh, Royal Edinburgh Hospital, Edinburgh EH10 5HF, UK

**Keywords:** environment, schizophrenia, major depressive disorder, genetics, gene–environment correlation

## Abstract

Research suggests that both genetic and environmental risk factors are involved in the aetiology of schizophrenia (SCZ) and major depressive disorder (MDD). Importantly, environmental and genetic risk factors are often related as evidenced in gene–environment correlation (rGE), which describes the observation that genetic and environmental factors are associated with each other. It is understood that rGE gets stronger over time as individuals select their environments more actively based on their genetic propensities. However, little is known whether rGEs remain stable over time or change across different development periods. Using data from three British longitudinal cohorts, we investigated whether rGE patterns of polygenic risk scores (PRS) for SCZ and MDD changed over time across childhood and adulthood, as well as across both from birth to age 55 and whether results differed between SCZ and MDD. Overall, the majority of rGEs remained stable across the investigated development periods. Furthermore, the few detected rGE changes which did differ between SCZ and MDD, could not be explained by the confounding of clinical cases and are therefore likely the result of actual changes in environmental and cultural risk factors with genetic susceptibility to SCZ and MDD likely playing a less significant role.

## 1. Introduction

A complex interplay between genes and the environment has been well established in the aetiology of psychopathologies [1,2]. One form of this intricate interplay is referred to as gene–environment correlation (rGE), which is traditionally understood to get stronger over time as individuals more actively select their own environments based on their genetic propensities [3,4]. Whilst few studies have explored rGE over time across different developmental periods [5], our study will address this gap by investigating the relationship between the genetic liability for schizophrenia (SCZ) or major depressive disorder (MDD) and correlated environmental risk factors over time as individuals develop more independence and shape their own environments. In this study, we aim to identify: (a) whether rGEs change over time across childhood, (b) whether rGEs change over time across adulthood, (c) whether there is a significant difference in the strength of rGE associations from child- to adulthood and (d) whether results differ between SCZ and MDD.

### 1.1. Prevalence of SCZ and MDD

The prevalence of SCZ and MDD with 1% and 16–20%, respectively, is well established in adults [6,7,8]; however, we know only little about these two psychopathologies, particularly SCZ, in children. This is primarily due to an age of onset in late adolescence or early adulthood for both disorders [9,10], thus making early diagnosis difficult. The limited number of epidemiological studies estimate a prevalence of childhood-onset SCZ between 1 in 30,000 to 1 in 40,000 children [11,12]. However, the prevalence of MDD in childhood and adolescence is slightly higher, ranging from 0.87% to 1.43% in pre-school children [13] to an overall prevalence of 0.4–2.8% in children and 0.4–8.3% in adolescents [14,15].

### 1.2. Genetic Influences

Both SCZ and MDD have substantial heritability estimates in adults with twin and family studies approximating ~80% [2,16] and ~38% [17,18] for SCZ and MDD, respectively. Whilst few studies have assessed the heritability of SCZ in children, Rutter et al. (2006) [19] suggest that the heritability of MDD is at a low level in children and increases to moderate levels in adolescents and then stays relatively stable until adulthood.

Further, the genetic architecture of both psychopathologies is highly polygenic, consisting of thousands of single nucleotide polymorphisms (SNPs) [20]. Although the effects of each single SNP are very small, these can be combined into Polygenic Risk Scores (PRS) consisting of the weighted sum of the risk-associated alleles to estimate the genetic propensities to these psychopathologies for each individual [21,22].

### 1.3. Environmental Influences

PRS-based methods are a useful tool to investigate gene–environment interplay given that SCZ and MDD are not just influenced by genetic but also by environmental risk factors, which themselves have been found to be heritable [2,20,23,24]. These environments can range from a short gestational period [25,26] and low parental educational attainment [27,28,29] in childhood, all the way to unemployment [23,30], low socio-economic status (SES) [31,32] and death of a spouse [33] in adulthood. However, environmental exposures are often influenced by individual behaviors, these behaviors will in turn often change as individuals transition from one developmental period to another. For instance, differences in temperament in early childhood lead to considerably bigger differences in antisocial behavior in adolescences [34]. Moreover, socio-cultural influences, such as changes in smoking behavior due to tobacco availability, further contribute to potentially differential exposures to environmental risks over time [35]. Nevertheless, studies need to focus not just on understanding the impact of the timing but also the effect of continuous adverse environmental exposures on the liability to psychopathology [36].

### 1.4. Psychopathology across Development

Early environmental exposures or events, including adversity and stress, can have lasting effects on our biology depending on the timing of these events during critical developmental windows [24,37]. Whilst the importance of a life-course perspective in psychiatric outcomes is generally well understood, investigating psychopathological outcome over time requires not just longitudinal data but also large samples [38]. Additionally, the divide between child and adult behavioral research and associated clinical services has often been a barrier to adopting a developmentally-focused approach [38]. Bearing in mind that half of all mental health lifetime cases occur by the age of 14 and 75% by 24 years, with late onset mental health outcomes often being a co-morbidity [39], this raises the question, not just what the environmental targets for treatments or preventions are, but also if these targets change as individuals transition from childhood to adulthood. This is further complicated by the heterogenous symptomology for some psychiatric disorders, such as for SCZ and MDD, during different developmental stages [9,10,19]. For instance, SCZ phenotypes range from autistic symptoms and cognitive disabilities in childhood [40,41] to anxiety in adolescents [42], whilst MDD often manifests itself as irritable mood, lack of weight gain and anhedonia in children [43], to subthreshold depressive symptoms in pre-adolescents [44].

### 1.5. rGE across Development

From a perspective of rGE, genetic risk variants can influence the *exposure* to environmental factors through either *passive* rGE, when parents pass their genes to their offspring whilst also shaping their environment; *active* rGE, whereby a genetically influenced behavior predicts the probability of exposure to an environmental factor; or *evocative* rGE when a genetic predisposition modulates an individual’s behavior which then evokes a response from others [4]. It has further been suggested that there is a developmental shift from passive rGE to evocative and active rGE occurring between infancy and adolescence as children start to more actively shape their environments [3], with active rGE being more prevalent later in life compared to evocative [45]. For instance, a recent study investigated the correlations of several socio-environmental factors, including urbanization, and the PRS for SCZ and MDD (amongst others) in 2232 British twins who were born between 1994–1995 and followed up until age 18 in the Environmental Risk Longitudinal Twin Study [46]. The study identified that there was some evidence to suggest that rGE increases across childhood, whereby associations between the PRS for SCZ and urbanicity and between the PRS for MDD and deprivation increased over time as the children got older [46].

### 1.6. The Current Study

We will be utilizing existing longitudinal data from three British non-clinical cohorts, the Millennium Cohort Study (MCS), Understanding Society (USoc) and the 1958 National Child Development Study (NCDS), to investigate whether previously detected rGE findings in these samples [47,48] change across childhood (from birth to age 16) and across adulthood (from 16 years or over onwards), separately for SCZ and MDD (see Table 1). Moreover, given that individuals from the NCDS study were followed from birth to 55 years of age, we will additionally investigate whether there is a significant change in the strength of previously detected rGE correlations between childhood to adulthood. Finally, due to the partial genetic overlap between the two psychopathologies, we will also test whether rGE changes over time differ between SCZ and MDD.

### 1.7. Hypotheses

Based on previous research findings [46], we hypothesized that previously identified rGE correlations between the PRS for SCZ or MDD and environmental risk factors would increase over time as individuals start to shape their own environments due to active rGE. Further, we expected that rGE associations would be stronger in adulthood compared to childhood, but that these would differ for SCZ and MDD given the incomplete genetic overlap between the two psychopathologies.

## 2. Methods

### 2.1. Participants

Individuals (males and females) were taken from three British Community cohorts. Firstly, 18,827 children from 18,552 families who were born between September 2000 and January 2002 in the United Kingdom (UK) from different ethnic backgrounds participated in MCS [49,50]. Parents, teachers and cohort members completed reports across six data waves with participants being 9 months of age in 2001 up until 14 years of age in 2015. This included the collection of 23,336 saliva samples for DNA extraction at wave 6 from the participating children and their biological parents [50,51,52].

Secondly, USoc is made up of approximately 40,000 households from the UK who were assessed annually through online reports or face-to-face interviews [53,54]. We only included participants who were aged 16 or over, who used the adult questionnaires [53] from nine waves starting in 2009/2010 (participants aged 16 to 97) until 2017/2018 (aged 22 to 104). Health assessments, including DNA samples from approximately 10,000 participants in USoc, were collected during data sweeps 2 and 3 [55].

Thirdly, 17,415 unrelated participants from NCDS were born in a single week in March 1958 in England, Wales or Scotland [56,57]. We had access to ten waves of the NCDS from 1958 (birth of participants) up until 2013 (participants aged 55). Initial childhood sweeps from birth to age 16 included midwife and clinical records as well as parent and teacher surveys [56]. Cohort members continued to be assessed in adulthood, which included a bio-medical survey and blood samples collection from 9293 participants for DNA extraction between 2002 and 2004 [58].

Cohort characteristics have been summarized in supplementary document S5.

### 2.2. Measures

#### 2.2.1. Environmental and Psychosocial Risk Factors

The current paper focuses on a selection of established environmental and psychosocial risk factors for SCZ and MDD that were significantly correlated with polygenic scores for SCZ or MDD (for at least one PRS threshold with a *p*-value of less than 0.05 prior to multiple testing and measured across multiple timepoints in childhood or adulthood) in our previous analyses of these data [47,48].

Results from our recent rGE childhood study [48] suggested that the PRS for SCZ and MDD were associated with parents being separated, divorced or widowed. Additionally, fathers not being involved in their offspring’s upbringing was correlated with the genetic susceptibility to SCZ. Moreover, an increased genetic propensity to MDD was correlated with several indicators of low SES, including low number of bedrooms and rented accommodation, lack of parental interest in the offspring’s education, increased maternal smoking and decreased maternal alcohol consumption. Our study suggested that more than half of these childhood correlations reflected passive rGE. Findings from our recent rGE adulthood [47] analysis highlighted an association between the genetic liability to SCZ and being single or divorced. Secondly, a higher PRS for MDD was associated with low income, financial issues, unemployment and several indicators of low SES, such as rented accommodation and low number of bedrooms.

Previously identified environmental variables that were only measured at a single timepoint were excluded from the current analysis. Furthermore, the childhood vs. adulthood rGE-by-time comparison analysis in the NCDS data only included significant environmental variables which were available both in childhood *and* in adulthood. All selected environmental and psychosocial risk factors are listed in Table 1 (for more detailed information on the individual variables see supplementary document S3).

#### 2.2.2. Genetic Data Processing and Polygenic Risk Scoring

We used genome-wide SNP data from 21,324 participants (8201 children and 13,123 biological parents) who were genotyped on Illumina’s Infinium global screening array 24 v1.0 [52] from MCS as well as 9961 participants from USoc genotyped on Illumina Infinium HumanCoreExome BeadChip array [59]. For NCDS, SNP data was available from three separate studies: 1502 individuals from the Wellcome Trust Case Control Consortium 1 (WTCCC1); 2592 participants from the Type 1 Diabetes Genetics Consortium (T1DGC); and 2922 participants from the Wellcome Trust Case Control Consortium 2 (WTCCC2) which were genotyped on Affymetix 500 k 1.2 M [60], Infinium Humanhap 550 k v3 [61] and Illumina 1.2 M [62], respectively.

Genetic quality control (QC) was performed for each cohort separately according to Coleman’s GWAS codebook [63] and has been described in detail elsewhere [47,48]. To summarize, using Plink 1.9 [64], for MCS only, the genetic data was first split into a child and adult dataset before calculating genome-wide identical-by-state (IBS) and clustering individuals into 14 homogenous groups which were then overlaid with references from the 1000 Genomes Project [65]. Individuals from clusters closest to European ancestry (7025 children and 11,269 biological parents) were used as a European subset, with all other clusters (1176 children and 1852 biological parents) merged into a non-European subset.

For all subsets from all three cohorts, we removed duplicated individuals, samples with missing data (<99%), minor allele frequencies (MAF < 1%), SNPs which deviate from Hardy–Weinberg Equilibrium (*p* ≤ 1 × 10^−5^), related individuals (pi-hat < 0.1875) and individuals with mismatching genetic and phenotype sex, before pruning for linkage disequilibrium (LD, r^2^ < 0.2). Additionally, ancestry outliers were excluded, heterozygosity was assessed (> or <3SD from mean) and reverse or ambiguous strand SNPs were identified using SNPFLIP v0.0.6 [66]. Only the NCDS data required a genome build liftover, from build 36 to 37 for WTCCC2 and T1DGC as well as build 35 to 37 for WTCCC1, using liftOverPlink [67] before submitting individual chromosome files for each subset and cohort for imputation to the Michigan imputation server [68]. Post-imputation QC included the removal of SNPs with low imputation quality (R2 > 0.8) and posterior genotype probability imputation confidence (GP threshold of > 0.8) using bcftools [69] as well the exclusion of duplicated, missing, or failed SNPs as well as MAFs (<5%) and individuals with <99% genotypes followed by combining each chromosome dataset into the final genotype file for each cohort. Subsets for MCS and NCDS were combined for each cohort and duplicates removed. Principal component analysis was repeated for the whole cohort dataset for MCS and NCDS.

PRS was computed for each participant from the 3 cohorts at seven thresholds in PRSice [70]. For MCS and USoc, we used GWAS results from the Psychiatric Genetics Consortium Working Groups for Schizophrenia [71] and Major Depressive Disorder [72]. As NCDS was used as a control by both working groups, we utilized updated GWAS which omitted UK (but not Irish) studies for SCZ and GenPod and 23andme for MDD.

#### 2.2.3. Data Analysis

Analyses were conducted for each cohort separately in Stata v12.1 [73]. Firstly, PRS scores were combined with the phenotype file for each cohort. For USC only, we randomly selected one individual from each household to obtain a sample of genetically unrelated participants from different families. The final datasets for our three cohorts were comprised of 7280 children (6874 mothers & 4322 fathers) from MCS; 7384 individuals from USoc; and 5288 participants from NCDS.

Secondly, for rGE changes over time in childhood we used data from the MCS (birth until age 14) and childhood data from NCDS (from birth to age 16). To identify any rGE changes over time in adulthood we utilized adult data from individuals over the age of 16 from USoc and adulthood data from NCDS (from age 23 to age 55). Additionally, for our rGE childhood vs. adulthood comparison, we used NCDS data from birth up to age 55.

Thirdly, in order to test whether rGEs differed over time in childhood or adulthood, we combined environmental risk factors at different timepoints into either logistic or linear mixed effects or random effects longitudinal models which were then fitted with full factorial two-way interactions between the PRS and time which were coded as continuous variables. For the childhood vs. adulthood analysis in NCDS, we coded the childhood and adulthood environments as binary variables (0 = childhood, 1 = adulthood) and added the PRS as well as the binary child-adulthood variable as two-way interactions into the mixed-effects or random effects regression models. Changes in the strength of rGE correlations were interpreted using the regression β coefficient (β), with a negative *β* suggesting a reduction in strength of rGE across time and a positive *β* indicating an increase in the strength of rGE over time. For MCS, all calculations were computed using the children’s PRS only. All rGE by time calculations included birth year (USoc), sex and the top 5, 4 and 8 principal components for MCS, USoc and NCDS, respectively, which explain the majority of the variance as covariates. All results (80 correlation calculations with 7 thresholds each, resulting in a total of 560 individual outputs) from the three community cohorts were corrected with the Benjamini–Hochberg correction: adjusted α = (rank of *p*-value/number of tests for each threshold) × α [adjusted α = (rank/560) × 0.05]. Findings were considered statistically significant if at least one PRS *p*-value threshold met the Benjamini–Hochberg correction (See supplementary document S4). 

Fourthly, sensitivity analyses were run for any significant findings after correction for multiple testing which included one significant SCZ finding for MCS in childhood, one SCZ and two MDD findings for the NCDS child vs. adulthood comparison, as well as two MDD findings for USoc in adulthood. For MCS, we re-ran the one significant finding by including the maternal and paternal PRS as covariates to assess whether passive rGE could contribute to change over time. Whilst we do not have the parental genotypes for USoc and NCDS, we wanted to exclude the likelihood of clinical cases confounding our findings. Therefore, for USoc, we excluded individuals who reported clinical depression (*n* = 448) and those treated for psychiatric problems (*n* = 111). Sensitivity analyses for the one significant USoc SCZ finding was not performed due to the lack of SCZ symptoms or diagnoses in the cohort. Similarly, for NCDS, 1397 individuals who self-reported depression in wave 9 (aged 55) were removed [74].

Moreover, we added an interaction between all independent variables and either the maternal/paternal PRS for MCS or the SCZ/MDD symptoms for USoc and NCDS using the resulting Wald Chi-squared test statistics if the β coefficients from our original regressions and sensitivity analyses are statistically different.

Full descriptive statistics for all three cohorts had been provided elsewhere [47,48] and power calculations (computed using G*Power v3.1 [75]) are described in supplementary document S1.

Heatmaps were created in R v3.5.0 [76].

## 3. Results

### 3.1. rGE across Childhood

**Figure 1:**
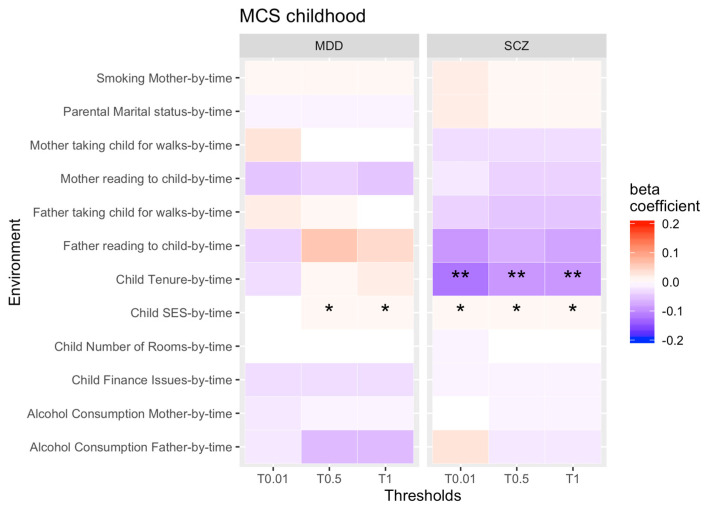
rGE changes across childhood (interaction terms PRS*time). Note: * = significant, ** = significant after correcting for multiple testing. Not all thresholds have been included (0.1, 0.2, 0.4, 0.4 have been omitted).

**Figure 2:**
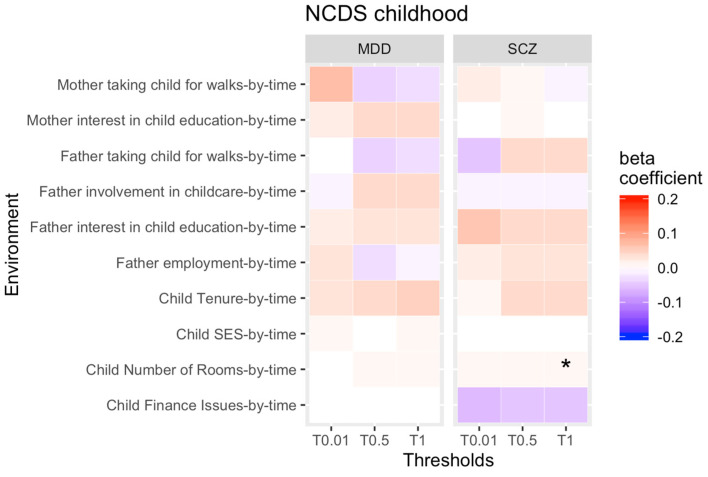
rGE changes across childhood (interaction terms PRS*time). Note: * = significant. Not all thresholds have been included (0.1, 0.2, 0.4, 0.4 have been omitted).

Our first aim was to test for rGE changes over time for each PRS threshold and each longitudinal environmental exposure across childhood (Figure 1 and Figure 2 display results for PRS thresholds 0.01, 0.5 and 1 only).

In MCS, the correlations between low SES and the genetic propensity for SCZ and MDD got stronger in childhood. In other words, as children became older, a higher genetic risk of both psychopathologies was more strongly correlated with low SES. Furthermore, the association between rented accommodation and the genetic susceptibility for SCZ got weaker in childhood, meaning the PRS for SCZ is less associated with tenure of accommodation as children grow older.

Moreover, the strength of the association between the genetic liability for SCZ and low number of bedrooms increased over time in NCDS, reflecting that the genetic risk of SCZ got more strongly associated with low number of bedrooms as children got older.

Overall, only the SCZ finding for rented accommodation in MCS survived the Benjamini–Hochberg correction. Our sensitivity analysis suggests that this finding was not confounded by parental genotypes.

All other rGE correlations remained stable across childhood and did not change.

### 3.2. rGE across Adulthood

**Figure 3:**
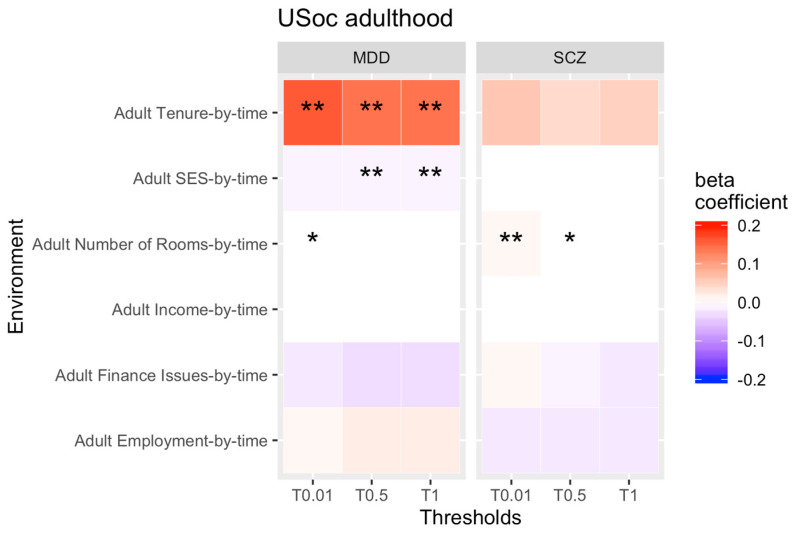
rGE changes across adulthood (interaction terms PRS*time). Note: * = significant, ** = significant after correcting for multiple testing. Not all thresholds have been included (0.1, 0.2, 0.4, 0.4 have been omitted).

**Figure 4:**
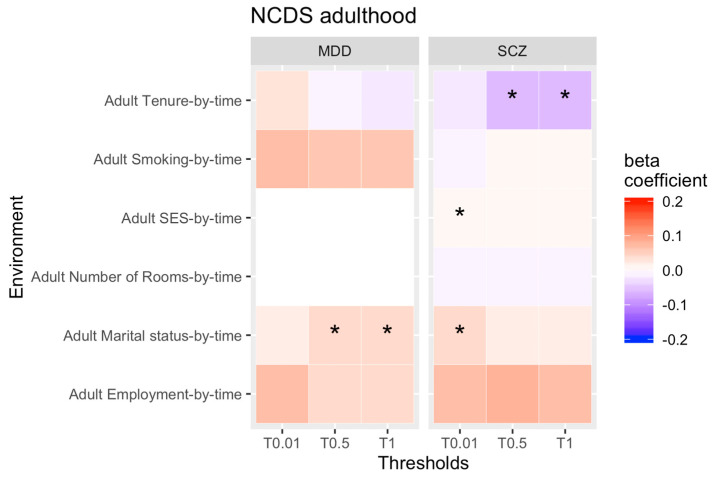
rGE changes across adulthood (interaction terms PRS*time). Note: * = significant. Not all thresholds have been included (0.1, 0.2, 0.4, 0.4 have been omitted).

Our second aim was to identify changes of rGE over time in adulthood (see Figure 3 and Figure 4 for all results for PRS thresholds 0.01, 0.5 and 1 only).

For USC, we found that the correlation between the genetic risk of SCZ and MDD and low number of bedrooms slightly increased across adulthood. In other words, higher genetic propensity for both psychopathologies became more strongly associated with low number of bedrooms as adults grow older. However, the effect sizes for these correlations were very small and only the SCZ finding continued to be significant after correction for multiple testing. In addition, we found that the association between the PRS for MDD and low SES became weaker over time, whilst the correlation between rented accommodation with the PRS for MDD increased over time. That means, as adults get older, a higher genetic risk of MDD becomes more strongly correlated with tenure of accommodation, but less with low SES. Both findings survived multiple testing. Our USoc sensitivity analysis suggests that the increased change between the genetic risk for MDD and tenure could be confounded by clinical cases.

For NCDS, we identified that the genetic propensity for SCZ and being single/divorced as well as the genetic liability for MDD and being in a relationship increased over time. That means that higher genetic risk of either psychopathology becomes more strongly associated with being single/divorced as adults age. On the other hand, the association between genetic susceptibility to SCZ and SES got stronger, whereas the correlation with rented accommodation decreased over time. Therefore, higher genetic risk of SCZ is more strongly associated with higher SES as individuals get older, but less correlated with tenure of accommodation, which gets weaker as individuals grow older. However, none of our findings for NCDS survived after correction for multiple testing.

All other rGEs were stable across adulthood for both cohorts.

### 3.3. Comparison between Childhood and Adulthood

**Figure 5:**
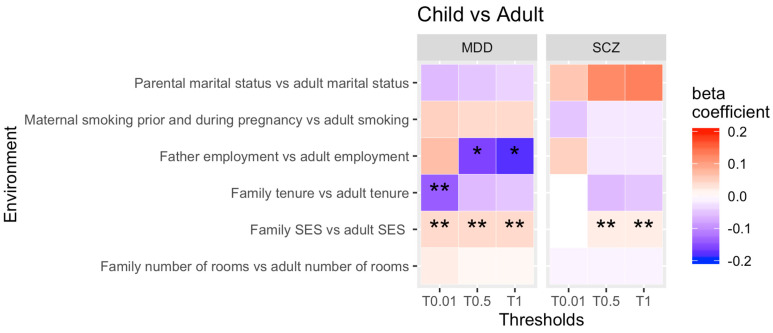
Childhood vs. adulthood comparison in NCDS. Note: * = significant, ** = significant after correcting for multiple testing. Not all thresholds have been included (0.1, 0.2, 0.4, 0.4 have been omitted).

Thirdly, we wanted to investigate whether there are significant changes in rGE associations between childhood and adulthood in individuals from NCDS only (see Figure 5 for all results for PRS thresholds 0.01, 0.5 and 1 only).

Our findings show that the strength of rGE between the genetic susceptibility to SCZ and higher SES was stronger in adulthood (family SES in childhood vs. adulthood SES), whilst the genetic risk of MDD and low SES also increased from child- to adulthood (family SES in childhood vs. adulthood SES). Furthermore, the rGE between unemployment and the genetic liability for MDD decreased over time, meaning that the association between the child’s genetic risk of MDD and the father’s unemployment was stronger in childhood compared to the individual’s unemployment status as an adult. Finally, the strength of rGE between the genetic susceptibility to MDD and rented accommodation (family home vs. adult home) decreased over time. That means that this correlation was stronger in childhood compared to adulthood.

All findings, but for the change in unemployment over time, survived the Benjamini–Hochberg correction. Our sensitivity analyses suggest that none of the findings were confounded by the presence of clinical cases. See supplementary document S4.

Similar to our individual childhood and adulthood rGE-by-time analysis, the majority of our rGE childhood vs. adulthood comparison in NCDS shows that most rGE remain stable across the two generations.

### 3.4. Sample Size and Power Calculation

According to our power calculation (See supplementary document S1), all three cohorts were sufficiently powered, except for SES at wave 8 and 9 in USoc, and in NCDS for father’s interest in the child’s education at ages 7, 11 and 16, employment at age 23 and tenure at age 23 and 55. Additionally, some differences emerged between the original samples and the final genetic subsamples for all three cohorts.

## 4. Discussion

The aim of this study was to investigate rGE changes across time in both childhood and adulthood involving a set of established environmental and psychosocial risk factors and polygenic scores for SCZ and MDD from three separate cohort studies. This study provides further advances on our two previous studies using the same data [47,48].

### 4.1. Change in rGE across Childhood

The current findings suggests that rGEs for SCZ and MDD appear to be relatively stable across childhood, except for tenure of accommodation for individuals with higher genetic risk of SCZ. In other words, there is no change in the strength for the majority of these associations between birth up until age 14 in MCS and from birth up until age 16 in NCDS. Although previous research has suggested that rGE may become stronger as individuals actively select themselves into environments which are corresponding to their genetic predisposition [3,4], our study suggests that this shift from passive rGE to evocative/active rGE does not yet occur in childhood. Whilst Newbury et al. (2020) identified rGE changes across different developmental windows for some socio-economic indicators which were correlated with the PRS for SCZ and MDD in children and adolescents between the ages of 12 and 18 years of age [46], we only obtained one significant finding whereby the strength of the association between the genetic risk of SCZ and rented accommodation became weaker as children grow older. Our sensitivity analysis suggests that this change over time is not due to passive rGE and therefore likely due to sociocultural changes in environmental risk, such as changes in homeownership due to financialization [77]. One reason that we did not obtain more findings of change in rGE could be the MCS and NCDS childhood samples only include individuals up to the age of 14 and 16, respectively. Additionally, it is also plausible, that environments for adolescents may have remained largely the same in the UK during this period. However, bearing in mind that children will undergo rapid changes during specific developmental periods, more detailed phenotypic and environmental measurements may be required to track these changes [34] than was available in our data. Moreover, it is also conceivable that our selected psychosocial indicators may be cohort specific [78] and therefore do not reflect universal rGE changes across childhood.

### 4.2. Change in rGE across Adulthood

In USoc we identified that the strength of the rGE association between the genetic predisposition to SCZ and low number of bedrooms during adulthood got stronger. This could be a reflection of increasing urbanization, which is a well-known environmental risk factor for SCZ [79]. However, we would also note that the effect size of this change is very small and therefore should be interpreted with caution. Whilst Jaffee et al. (2007) suggest that environments and genes can have time-dependent effects on the aetiology of psychopathological outcomes [3], it is not inconceivable that our findings are due to generational changes in environmental risk (cohort effect) as our result is specific to USoc only which started in 2009 and has not been found in NCDS where individuals were born in 1958.

In addition, our results show that the effects of the PRS for MDD on established environmental risk factors only changed for SES and tenure of accommodation across adulthood. In more detail, the association between the genetic risk of MDD and low SES gets weaker as individuals shape their own environments through active rGE. In contrast, all other rGE associations remain relatively constant in individuals from USoc (mixed ages) from 16 years onwards and in participants from NCDS from the age of 23 years until age 55. Although, the association between the PRS for MDD and rented accommodation became stronger over time in adulthood, it is also important to highlight that this finding is confounded by the presence of individuals with clinical depression as per our sensitivity analysis.

Overall, the strength of rGE did not change over time for the majority of environmental risk factors. However, larger sample sizes may be better able to detect changes [38].

### 4.3. Childhood vs. Adulthood PRS-by-Time Comparison

Whilst the strength of rGE association between SES (family SES in childhood vs. individual’s SES in adulthood) and the genetic propensity for SCZ and MDD got stronger as individuals moved from childhood to adulthood, in contrast to our hypothesis, the strength of rGE between the genetic risk SNPs for MDD and rented accommodation (family tenure in childhood vs. individual’s tenure in adulthood) were stronger in childhood compared to adulthood. It is possible, that transgenerational transmission may play a confounding role in rGE being stronger in childhood [80]. Shared genetic inheritance between the biological parents and their offspring, in addition to providing their children with a family environment in line with their genetic predisposition, may result in similarities in their behavior across generations. However, in contrast to our findings, results from one Dutch twin study, which looked into the association between depressive symptoms and well-being, suggests that the role of genetic effects in comparison to environmental influences get stronger from adolescence onwards [81]. Considering that individuals from NCDS were born in 1958 and would have been young adults in the 1980s, one possible explanation for the change in environmental risk is the UK Housing Act under the Thatcher legislation which aimed to increase home ownership [82]. However, it is also important to note that the development of MDD from childhood to adolescence and then into adulthood is complex with ever changing environmental and genetic influences. Although our childhood versus adulthood analysis was looking at rGE changes from birth until age 55, we do not have any data points between the ages of 16 and 23 years in order to differentiate between all stages of development. Moreover, environmental risk in childhood refers to the family or parent environments, whereas in adulthood, it is the individual themselves and therefore may not be sufficiently comparable. Our analysis should therefore be deemed exploratory and should encourage future studies to look at rGE associations across different developmental periods.

### 4.4. Comparison between SCZ and MDD

Our last objective was to compare if rGE changes over time differed between SCZ and MDD. Indeed, results for SCZ and MDD did not match between childhood and adulthood. We found one significant change in rGE over childhood for SCZ (tenure of accommodation) but none for MDD. Additionally, we identified only one change in rGE over adulthood (number of bedrooms) in SCZ, but two additional rGE by time changes (SES and tenure) for MDD. Moreover, when comparing childhood and adulthood environments in NCDS, we only obtained one match for SCZ and MDD rGE changes over time for SES, whereby the rGE strength for tenure only changed for MDD between childhood and adulthood. Whilst some of these mismatches could be explained by the incomplete genetic overlap between the two disorders, it is also likely that generational changes in environmental risk are at least partially contributing to these findings.

### 4.5. Strengths and Limitations

Whilst one of the strengths of our study is being able to investigate rGE changes across two different developmental periods in three different well-powered generational cohorts, we would also like to highlight some limitations. Firstly, our study only investigated environmental risks which were previously identified as being associated with the genetic liability to either SCZ or MDD in two of our previous analyses of the same data [47,48]. Therefore, findings differed between the three community cohorts for several reasons: not all environmental measures are matching across all three samples. Further, our study is using longitudinal data from three British community cohorts where data was collected at different intervals and at different ages. In addition, our childhood versus adulthood comparison analysis in NCDS lacked the ability to differentiate between all early developmental stages (late childhood and adolescence) as no data was available between the ages of 16 and 23. Moreover, the USoc cohort included individuals of mixed ages (aged 16 to 97 at wave 1) whereas individuals from NCDS (birth to age 55) and MCS (9 months to 14 years of age) were all born in the same week or birth year, respectively. Further, our descriptive statistics suggests that we do not have sufficient power for some environmental measures and that there are some differences between the genetic subsample and the three community cohorts. Finally, future studies may also want to investigate the role of gender differences in the stability of rGE.

### 4.6. Implications

Although the genetic risk of SCZ and MDD does not change across the life course, behaviors which affect exposure to environments change as we transition through different developmental periods, with the impact of these exposures also depending on whether these occur in critical periods of development [34,37]. Despite the gene–environment interplay between the PRS for SCZ and MDD, and some of our identified environmental and psychosocial risk factors appearing to vary across different development periods, translating these findings into a clinically relevant context is still challenging [83]. Nevertheless, it is important to point out that rGE does not exclude the possibility that environmental exposures may also have causal or reciprocal consequences on complex psychopathologies, and if this is the case, then we need to better understand the extent of those influences to effectively assess targets for treatments or interventions [34]. To put this into context, there are few rGE changes over time and the effect sizes for our findings were very small and therefore would have little impact overall across the general population. Moreover, our results are likely due to additional changes in environmental and cultural risk over the decades, meaning that the genetic susceptibility to SCZ and MDD would have little impact.

## 5. Conclusions

Using data from three large British community cohorts, we investigated whether established rGE change over time for SCZ and MDD. Our findings suggest that rGE for SCZ and MDD remained largely stable in childhood and did not change considerably, except for one marker of low SES for SCZ. Moreover, the strength of rGEs in adulthood only changed for one indicator of urbanization for SCZ and two markers of low SES for MDD. Moreover, by investigating rGE changes between childhood and adulthood in NCDS, we showed that the genetic liability to SCZ and MDD on SES increased across the life course for both psychopathologies, as well as decreasing for rented accommodation for MDD. However, effect sizes for all significant findings were small and therefore our findings must be interpreted with caution. Finally, results for SCZ and MDD did not match, suggesting that rGE changes over time are likely disorder specific.

## Figures and Tables

**Table 1:** Selected environmental risk factors.

Analysis	Economic Situation	Substance Abuse	Psychosocial Outcomes
Childhood rGE by time analysis	-SES, -tenure, -financial issues, -number of bedrooms, -employment	-maternal smoking, -maternal alcohol consumption, -paternal alcohol consumption	-parental marital status, -father’s involvement in the child’s upbringing, -maternal interest in the child’s education, -paternal interest in the child’s education, -mother takes child for walks, -father takes child for walks, -mother reads to child, -father reads to child
Adulthood rGE by time analysis	-SES, -tenure, -financial issues, -number of bedrooms, -employment, -income	-smoking	-marital status
Childhood vs. adulthood rGE by time analysis	-family SES in childhood vs. SES of individual in adulthood, -family tenure in childhood vs. tenure of individual in adulthood, -family number of bedrooms in childhood vs. number of bedrooms of individual in adulthood, -father’s employment in childhood vs. employment of individual in adulthood	-mother’s smoking behaviour prior and during pregnancy vs. smoking behaviour of individual during adulthood	-marital status of mother at birth vs. marital status of individual in adulthood

Note: Any significant findings from [47,48] which were not available at multiple timepoints were excluded from our rGE across time analysis.

## Data Availability

The datasets used to support the findings of this study are available from the Centre for Longitudinal Studies (https://cls.ucl.ac.uk/cls-studies/) and the UK Data Services (https://ukdataservice.ac.uk).

## Supplementary Materials

The following supporting information can be downloaded at: https://www.mdpi.com/article/10.3390/genes13071136/s1, Supplementary Document S1—Power calculation; Supplementary Document S2—Coded variables; Supplementary Document S3—environmental risk factors; Supplementary Document S4—SCZ and MDD results, Supplementary Document S5—Cohort_characteristics.
