# Supplementary material for: Gene–Environment Correlation over Time: A Longitudinal Analysis of Polygenic Risk Scores for Schizophrenia and Major Depression in Three British Cohorts Studies
Source: Genes (Basel). 2022 Jun 24;13(7):1136. doi: 10.3390/genes13071136 (PMC9320197; doi:10.3390/genes13071136)
Supplement: Supplementary file 1 [file genes-13-01136-s001.zip › Supplementary_S1_Power_Calculation.pdf]

Supplementary Document S1– Power Calculation

Table S1 : Power Calculation - MCS

Power calculation model used	F-test (Fixed linear regression)
Power	0.8
Effect size	0.005
Error rate	0.0021 (=0.05/24 environments tested)
Number of tested predictors	1
**Minimum sample size required**	**3,074**

Table S2 : Power Calculation – USoc

Power calculation model used	F-test (Fixed linear regression)
Power	0.8
Effect size	0.005
Error rate	0.0042 (=0.05/12 environments tested)
Number of tested predictors	1
**Minimum sample size required**	**2,760**

Table S3: Power Calculation - NCDS

Power calculation model used	F-test (Fixed linear regression)
Power	0.8
Effect size	0.005
Error rate	0.00111 (=0.05/44 environments tested)
Number of tested predictors	1
**Minimum sample size required**	**3,376**

Summary

For MCS, we identified an increased percentage of mothers who smoked at wave 1, decreased percentage of parents who were married at all waves, higher percentage of participants who were recorded as SES class 1 at waves 1, 3, 4 and an increased number of families who rented their homes in wave 6 in the genetic subsample.

For USoc, individuals from our genotype subsample had a lower number of bedrooms at wave 1-9 and decreased odd of being married at wave 1 compared to the whole cohort.

Moreover, individuals from the NCDS genotype sample were more likely to live in rented accommodation at age 7 compared to the whole cohort.

All Power calculations were calculated using G*Power 3.1 [1].

References

1. Faul, F., et al., *Statistical power analyses using G*Power 3.1: tests for correlation and regression analyses.* Behav Res Methods, 2009. **41**(4): p. 1149-60.
