# Supplementary material for: Gene–Environment Correlation over Time: A Longitudinal Analysis of Polygenic Risk Scores for Schizophrenia and Major Depression in Three British Cohorts Studies
Source: Genes (Basel). 2022 Jun 24;13(7):1136. doi: 10.3390/genes13071136 (PMC9320197; doi:10.3390/genes13071136)
Supplement: Supplementary file 1 [file genes-13-01136-s001.zip › Supplementary_S2_Coded_variables.pdf]

Supporting Document S2 – Coded Variables

Table of Contents

Table S4: Breakdown of variables for MCS (childhood) 2
Table S5: Breakdown of variables for USoc (adulthood) 6
Table S6: Breakdown of variables for NCDS (childhood) 10
Table S7: Breakdown of variables for NCDS (adulthood) 12
References 14

All variables were coded in STATA v12.1 (StataCorp, 2011).

Table S4: Breakdown of **variables** for MCS (childhood)

Environment Name	Variable		Coded as	Wave						Interaction Model	
	**Code**	**Name**		**1**	**2**	**3**	**4**	**5**	**6**	**Mixed effect**	**Random effect**
Alcohol mother	APALDR00	Frequency of alcohol consumption per week	0 = 1-2 times per month, Less than once a month, Never<br>1 = 5-6 times per week, 3-4 times per week, 1-2 times per week<br><br>Only coded mother's responses	X							X
	CPALDR00	Frequency of alcohol consumption per week				X					
	DPALDR00	Frequency of alcohol consumption per week					X				
	EPALDR00	Frequency of alcohol consumption per week						X			
	FPALDR00	Frequency of alcohol consumption per week							X		
Alcohol father	APALDR00	Frequency of alcohol consumption per week	0 = 1-2 times per month, Less than once a month, Never<br>1 = 5-6 times per week, 3-4 times per week, 1-2 times per week<br><br>Only coded father's responses	X						X	
	CPALDR00	Frequency of alcohol consumption per week				X					
	DPALDR00	Frequency of alcohol consumption per week					X				
	EPALDR00	Frequency of alcohol consumption per week						X			
	FPALDR00	Frequency of alcohol consumption per week							X		
Finance Issues	APMAFI00	How well respondent manages financially (self-rated)	0 = Living comfortably, doing alright, just about getting by<br>1 = finding it quite difficult, finding it very difficult	X						X	
	BPMAFI00	How well respondent manages financially (self-rated)			X						

	FPMAFI00	How well respondent manages financially (self-rated)	Coded mother's responses, if unavailable, used father's responses						X		
Parent's marital status	APFCIN00	Current legal marital status	0 = Married, 1st and only marriage, Remarried, 2nd or later marriage, Civil Partner (legally recognised)<br>1 = Legally separated, Divorced, Widowed, former Civil Partner, Surviving Civil Partner<br><br>Coded mother's responses, if unavailable, used father's responses	X							X
	CPFCIN00	Current legal marital status				X					
	DPFCIN00	Current legal marital status					X				
	EPFCIN00	Current legal marital status						X			
	FPFCIN00	Current legal marital status							X		
Mother reads to child	CPREOF0	How often do you read to CM	0 = Every day, Several times a week, Once or twice a week<br>1 = Once or twice a month, Less often, Not at all<br><br>Coded mother's responses only			X				X	
	DPREOF00	How often do you read to CM					X				
Father reads to child	CPREOF0	How often do you read to CM	0 = Every day, Several times a week, Once or twice a week<br>1 = Once or twice a month, Less often, Not at all			X				X	
	DPREOF00	How often do you read to CM					X				

			Coded father's responses only								
Rooms	APROMA00	Number of rooms - grouped	Continuous variable<br><br>Coded mother's responses, if unavailable, used father's responses	X						X	
	BPROMA00	Number of rooms - grouped			X						
	CPROMA00	Number of rooms - grouped				X					
	DPROMA00	Number of rooms - grouped					X				
	FPROMA00	Number of rooms (excl bath/toilets/halls)							X		
SES	ADD05C00	DV NS-SEC 5 classes (last known job)	1 = Semi-routine and routine<br>2 = Lower supervisory and technical<br>3 = Small employers and self-employed<br>4 = Intermediate<br>5 = Managerial and professional<br><br>Coded mother's responses, if unavailable, used father's responses	X						X	
	BDD05S00	Respondent NS-SEC 5 classes			X						
	CDD05C00	DV NS-SEC 5 classes (last known job)				X					
	DDD05C00	DV NS-SEC 5 classes (last known job)					X				
Smoking Mother	APSMUS0A	Current smoking MC1	0 = No, does not smoke<br>1 = Yes, cigarettes, Yes, roll-ups, Yes, cigars, Yes, a pipe, Yes, other tobacco product<br><br>Coded mother's responses only	X							X
	EPSMUS0A	Current use of tobacco products						X			
	FPSMUS0A	Current use of tobacco products							X		

Tenure	ADROOW00	Tenure of current home (owns/rents)	0 = Own outright, Own - mortgage/loan, Part rent/part mortgage (shared equity)<br>1 = Rent from local authority, Rent from Housing Association, Rent privately, Living with parents, Live rent free, Other<br><br>Coded mother's responses, if unavailable, used father's responses	X						X	(X for Sensitivity analysis)
	BDROOW00	Tenure of current home (owns/rents)			X						
	CDROOW00	Tenure of current home (owns/rents)				X					
	DPROOW00	Tenure of current home (owns/rents)					X				
	EPROOW00	DV Housing Tenure						X			
	FDROOW00	S6 DV Housing Tenure							X		
Mother walks child	CPWALK00	Frequency take child to park or playground	0 = Every day, Several times a week, Once or twice a week<br>1 = Once or twice a month, Less often, Not at all<br><br>Coded mother's responses only			X				X	
	DPWALK00	Frequency take child to park or playground					X				
Father walks child	CPWALK00	Frequency take child to park or playground	0 = Every day, Several times a week, Once or twice a week<br>1 = Once or twice a month, Less often, Not at all<br><br>Coded father's responses only			X				X	
	DPWALK00	Frequency take child to park or playground					X				

Note: Any 'N/A', 'Don't know', 'Blanks', 'Refusals', 'Can't say' or 'other' answers have been excluded from the analysis. SES = Socio-Economic Status

Table S5: Breakdown of **variables** for USoc (adulthood)

Environment	Variable		Coded as	Wave									Interaction Model	
	**Code**	**Name**		**1**	**2**	**3**	**4**	**5**	**6**	**7**	**8**	**9**	**Mixed Effect**	**Random Effect**
**Number of Rooms**	a_hsbeds	number of bedrooms (top-coded)	Continuous variable	X									X	
	b_hsbeds	number of bedrooms (top-coded)			X									
	c_hsbeds	number of bedrooms (top-coded)				X								
	d_hsbeds	number of bedrooms (top-coded)					X							
	e_hsbeds	number of bedrooms (top-coded)						X						
	f_hsbeds	number of bedrooms (top-coded)							X					
	g_hsbeds	number of bedrooms (top-coded)								X				
	h_hsbeds	number of bedrooms (top-coded)									X			
	i_hsbeds	number of bedrooms (top-coded)										X		
**SES**	a_jbrgsc_dv	Current job: Registrar General's Social Class	5 = Professional<br>4 = Managerial-technical<br>3 = Skilled non-manual and manual<br>2 = Partly skilled<br>1 = Unskilled	X									X	
	b_jbrgsc_dv	Current job: Registrar General's Social Class			X									
	c_jbrgsc_dv	Current job: Registrar General's Social Class				X								
	d_jbrgsc_dv	Current job: Registrar General's Social Class					X							

	e_jbrgsc_dv	Current job: Registrar General's Social Class						X						
	f_jbrgsc_dv	Current job: Registrar General's Social Class							X					
	g_jbrgsc_dv	Current job: Registrar General's Social Class								X				
	h_jbrgsc_dv	Current job: Registrar General's Social Class									X			
	i_jbrgsc_dv	Current job: Registrar General's Social Class										X		
**Income**	a_fimngrs_dv	total monthly personal income gross - 50-iles	Grouped into 50 sub-groups – 2% per group (total 100%)	X									X	
	b_fimngrs_dv	total monthly personal income gross - 50-iles			X									
	c_fimngrs_dv	total monthly personal income gross - 50-iles				X								
	d_fimngrs_dv	total monthly personal income gross - 50-iles					X							
	e_fimngrs_dv	total monthly personal income gross - 50-iles						X						
	f_fimngrs_dv	total monthly personal income gross - 50-iles							X					
	g_fimngrs_dv	total monthly personal income gross - 50-iles								X				
	h_fimngrs_dv	total monthly personal income gross - 50-iles									X			
	i_fimngrs_dv	total monthly personal income gross - 50-iles										X		
**Employment**	a_jbstat	Current economic activity	0 = employed/retired/ maternity leave/ apprenticeship<br>1 = unemployed/education/	X										X
	b_jbstat	Current economic activity			X									
	c_jbstat	Current economic activity				X								

	d_jbstat	Current economic activity	sick/in care/unpaid/gov training				X							
	e_jbstat	Current economic activity						X						
	f_jbstat	Current economic activity							X					
	g_jbstat	Current economic activity								X				
	h_jbstat	Current economic activity									X			
	i_jbstat	Current economic activity										X		
**Financial situation**	a_finnow	Subjective financial situation - current	0 = Comfortable financially and just getting by financially<br>1 = Experiencing financial difficulties	X										
	b_finnow	Subjective financial situation - current			X									
	c_finnow	Subjective financial situation - current				X								
	d_finnow	Subjective financial situation - current					X							
	e_finnow	Subjective financial situation - current						X						X
	f_finnow	Subjective financial situation - current							X					
	g_finnow	Subjective financial situation - current								X				
	h_finnow	Subjective financial situation - current									X			
	i_finnow	Subjective financial situation - current										X		
**Tenure**	a_hsownd	house owned or rented	0 = owner/shared ownership /mortgaged<br>1 = rented/ rent-free	X										X
	b_hsownd	house owned or rented			X									

	c_hsownd	own accommodation				X								
	d_hsownd	house owned or rented					X							
	e_hsownd	own accommodation						X						
	f_hsownd	house owned or rented							X					
	g_hsownd	house owned or rented								X				
	h_hsownd	house owned or rented									X			
	i_hsownd	house owned or rented										X		
**Depression**	a_hcond17	Clinical depression	0 = no<br>1 = yes	X									Used for Sensitivity Analysis	
	c_hcond17	Clinical depression				X								
	e_hcond17	Clinical depression						X						
	f_hcond17	Clinical depression							X					
	g_hcond17	Clinical depression								X				
	h_hcond17	Clinical depression									X			
	i_hcond17	Clinical depression										X		
**Psychiatric problems**	bk_hlprxi	Received treatment for psychiatric problems	0 = no<br>1 = yes	Individuals who previously participated in the British Household Panel study (now USoc) and received treatment for psychiatric problems during that time									Used for Sensitivity Analysis	
	bp_hlprxi	Received treatment for psychiatric problems												

Note: Any ‘N/A’, ‘Don’t know’ or ‘other’ answers have been excluded from the analysis. SES = Socio-Economic Status

Table S6: Breakdown of **variables** for NCDS (childhood)

Environment Name	Variable		Coded as	Age of participant				Interaction model	
	Code	Name		0	7	11	16	Mixed effect	Random effect
**SES**	N492	0 Social class mother's husband (GRO 1951)	5 Professional<br>4 Managerial/ Technical<br>3 Skilled<br>2 Partly-skilled<br>1 Unskilled	X				X	
	N190	1P Social class of father, male head (GRO 1960)			X				
	N1687	2PD Social class of father or male head (GRO 1966)				X			
	N1687	2PD Social class of father or male head (GRO 1966)					X		
**Finance Issues**	N315	1P Family difficulties -Financial	0 = No<br>1 = Yes		X			X	
	N1230	2P Serious financial hardship last yr				X			
	N2441	3P Serious financial trouble last yr					X		
**Number of Rooms**	N201	1P Number of rooms in household	Continuous variable		X			X	
	N1156	2P Number of rooms in accommodation				X			
	N1156	2P Number of rooms in accommodation					X		
**Tenure**	N200	1P Tenure of accommodation	0 = owns<br>1 = rents (including social housing)		X			X	
	N1152	2P Tenure of accommodation				X			
	N2471	3P Type of accommodation					X		
**Mother's interest in child's education**	N43	1S Mother's interest in childs education	0 = Interested (including some interest)<br>1 = Not interested		X			X	
	N852	2S Mothers' interest in childs education				X			

	N2325	3S Mother's interest in chlds education					X		
**Father's interest in child's education**	N44	1S Father's interest in childs education	0 = Interested (including some interest)<br>1 = Not interested		X			X	
	N851	2S Fathers' interest in childs education				X			
	N2324	3S Father's interest in chlds education					X		
**Father's involvement in childcare**	N183	1P Dads role in management of child	0 = Involved (including some involvement)<br>1 = Not involved		X			X	
	N1147	2P Dads role in management of child				X			
**Mother walks child**	N181	1P Outings with mother	0 = Most weeks/occasionally<br>1 = Hardly ever		X			X	
	N1145	2P Does mum take child for walks,visits				X			
**Father walks child**	N182	1P Outings with father	0 = Most weeks/occasionally<br>1 = Hardly ever		X			X	
	N1146	2P Does dad take child for walks,visits				X			
**Employment father**	N188	1P Unemployed,sick and retired (GRO 1960)	0 = Employed (including retired)<br>1 = Unemployed/sick		X				X
	N1172	2P Father,male head's occupation				X			
	N2383	3P Father or father figure's occupation (GRO 1970)					X		
**Maternal Smoking prior pregnancy**	N502	0 Smoking prior to pregnancy	0 = Non-Smoker<br>1 = Smoker	X				Used for childhood vs adulthood comparison	
**Maternal Smoking during pregnancy**	N503	0 Smoking during pregnancy	0 = Non-Smoker<br>1 = Smoker	X					
**Schizophrenia**	N9EMOP05	Type of Emotional Problem - Schizophrenia	0 = No<br>1 = Yes					Used for sensitivity analysis	
**Hallucinations**	N9EMOP04	Type of Emotional Problem - Hallucinations	0 = No<br>1 = Yes						
**Psychosis**	N9EMOP07	Type of Emotional Problem - Psychosis	0 = No<br>1 = Yes						

**Depression**	N9EMOP01	Type of Emotional Problem - Depression	0 = No<br>1 = Yes					

Note: Any 'N/A', 'Don't know' or 'other' answers have been excluded from the analysis. SES = Socio-Economic Status

Table S7: Breakdown of **variables** for NCDS (adulthood)

**Environment**	**Variable**		**Coded as**	**Variable timeline (Age of participant)**						**Interaction Model**	
	**Code**	**Name**		**23**	**33**	**42**	**46**	**50**	**55**	**Mixed Effect**	**Random Effect**
**SES**	N6149	4I Current or last job 1980 social class	5 Professional<br>4 Managerial/ occupations<br>3 Skilled<br>2 Partly-skilled<br>1 Unskilled	X						X	
	N540056	CASOC2:2 A4a/b) CURRENT/LAST JOB: RGs Social Class 1981			X						
	SC	(Current Job) Social Class				X					
	N7SC	(Derived) Social Class (RGSC SC based on Occ 1990)					X				
	N8SC	[SC2] Curr Job: Social Class (RGSC SC based on Occ 1990)						X			
	N9CSC	Social class 1990 based on soc2010 (CM current job)							X		
**Employment**	N4755	4I Whether currently unemployed	0 = Employed or Self-employed (full or part-time)<br>1 = Unemployed, sick, disabled<br>Removed retired	X						X	
	ECONACT	CMs current main activity				X					
	N8ECON02	[ECONACT2] (Recoded) CM's current economic activity						X			
	ND9ECACT	(Derived) Current economic activity status							X		
	N5323	4I Number of bedrooms	Continuous variable	X						X	

**Number of Rooms**	N502947	CMI:57 D9 No. rooms (apart from the bathroom & kitchen)			X						
	BEDROOMS	Number of bedrooms in current acco				X					
	N7NUMRMS	Number of rooms in the house					X				
	ND8NUMRM	(Derived) Number of rooms in the house (n8numrms)						X			
	N9NUMRMS	Number of rooms in home							X		
**Tenure**	N5333	4I Whether owner or renter	0 = owns/part-owns 1 = rents (including social housing)	X						X	
	TENURE91	DV:Housing tenure in 1991			X						
	TENURE2	Is current accom owned or rented				X					
	N7TEN	Home ownership / tenure status					X				
	N8TEN	[TENURE] Home ownership / tenure status						X			
	N9TEN	Whether CM owns or rents home or some other arrangement							X		
**Marital status**	PARTSTAT	4D Current partnership status	0 = married/ co-habiting/ in relationship 1 = not in relationship	X						X	
	N502549	CMI:51 C52 CM is cohabiting/married other			X						
	DMSPPART	Whether CM had current partner in hhld in NCDS V (FF)				X					
	N7MS12	Person's marital status - 02					X				
	ND8SPPHH	(Derived) Cohort member lives with a spouse or partner						X			
	ND9COHAB	(Derived) Whether CM cohabiting as a couple							X		
**Smoking**	CURRENTN	4D Smoking patterns	0 = Never smoked 1 = Smoker/ Ex-smoker	X						X	
	SMOKING	CM current smoking status				X					
	N8SMOKIG	[SMOKING] Smoking frequency						X			

Note: Any ‘N/A’, ‘Don’t know’ or ‘other’ answers have been excluded from the analysis. SES = Socio-Economic Status

References

StataCorp. (2011). *Stata Statistical Software: Release 12*. In College Station, TX: StataCorp LP.
