# Supplementary material for: Gene–Environment Correlation over Time: A Longitudinal Analysis of Polygenic Risk Scores for Schizophrenia and Major Depression in Three British Cohorts Studies
Source: Genes (Basel). 2022 Jun 24;13(7):1136. doi: 10.3390/genes13071136 (PMC9320197; doi:10.3390/genes13071136)
Supplement: Supplementary file 1 [file genes-13-01136-s001.zip › Supplementary_S3_environmental_factors.pdf]

Supplementary Document S3– Selected environmental risk factors

Table S8: Selected environmental risk factors which were correlated with the genetic risk for SCZ or MDD in Machlitt-Northen et al 2022 [1, 2]

Analysis	Significant Environmental risk factor	Used in MCS	Used in NCDS	Used in USoc
Childhood rGE by time analysis	SES[2]	Yes	Yes	N/A
	Finance Issues[1]	Yes	Yes	
	Number of Rooms[2]	Yes	Yes	
	Tenure[2]	Yes	Yes	
	Smoking Mother[2]	Yes	Variable unavailable	
	Alcohol consumption Mother[2]	Yes	Variable unavailable	
	Alcohol consumption Father[1]	Yes	Variable unavailable	
	Marital status[2]	Yes	No longitudinal data	
	Mother takes child for walks[1]	Yes	Yes	
	Father takes child for walks[1]	Yes	Yes	
	Alcohol Mother[1]	Yes	Variable unavailable	
	Employment[1]	No longitudinal data	Yes	
	Mother's interest in child's education[2]	No longitudinal data	Yes	
	Father's involvement in childcare[2]	No longitudinal data	Yes	
	Father's interest in child's education[2]	No longitudinal data	Yes	
	Mother reads to child[1]	Yes	No longitudinal data	
	Father reads to child[1]	Yes	No longitudinal data	
	birthweight[1]	No longitudinal data	No longitudinal data	

	Mother's age[1]	No longitudinal data	No longitudinal data	
	Housing Issues[1]	No longitudinal data	No longitudinal data	
	Domestic tension[1]	No longitudinal data	No longitudinal data	
Adulthood rGE by time analysis	SES[1]	N/A	Yes	Yes
	Number of Rooms[2]		Yes	Yes
	Tenure[2]		Yes	Yes
	Finance Issues[2]		Variable unavailable	Yes
	Marital status[2]		Yes	Yes
	Employment[2]		Yes	Yes
	Income[2]		Variable unavailable	Yes
	Smoking[1]		Yes	Variable unavailable
Childhood vs adulthood rGE by time analysis	Family SES in childhood vs SES of individual in adulthood	N/A	Yes	N/A
	Father's employment in childhood vs employment of individual in adulthood		Yes	
	Family number of bedrooms in childhood vs number of bedrooms of individual in adulthood		Yes	
	Family tenure in childhood vs tenure of individual in adulthood		Yes	
	Marital status of mother at birth vs marital status of individual in adulthood		Yes	
	Mother's smoking behaviour prior and during pregnancy vs smoking behaviour of individual during adulthood		Yes	

Note: [1] identified as significant environmental risk factor in Machlitt-Northen et al 2022 [1, 2] [2] identified as significant environmental risk factor after multiple testing in Machlitt-Northen et al 2022 [1, 2]. Any significant environmental risk factors which correlated with the genetic risk for SCZ or MDD from study 1 or 2 which were available as childhood and adulthood measures in NCDS were selected for the childhood vs adulthood rGE by time comparison.

References

1. Machlitt-Northen, S., et al., Polygenic Scores for Schizophrenia and Major Depression are Associated with Psychosocial Risk Factors in Children: Evidence of Gene-Environment Correlation [Accepted/In press] In: Journal of Child Psychology and Psychiatry. 2022.
2. Machlitt-Northen, S., et al., Polygenic Risk Scores for Schizophrenia and Major Depression are associated with Socio-Economic Indicators of Adversity in two British Community Samples [Manuscript submitted for publication]. 2022, Department of Biological and Experimental Psychology, Queen Mary University of London.
