# Supplementary material for: Gene–Environment Correlation over Time: A Longitudinal Analysis of Polygenic Risk Scores for Schizophrenia and Major Depression in Three British Cohorts Studies
Source: Genes (Basel). 2022 Jun 24;13(7):1136. doi: 10.3390/genes13071136 (PMC9320197; doi:10.3390/genes13071136)
Supplement: Supplementary file 1 [file genes-13-01136-s001.zip › Supplementary_S4_SCZ_and_MDD_results.pdf]

Supplementary Document S4– Full SCZ and MDD results

Table of Contents

Table S9: All PRS results for MCS 2
Table S10: All PRS results for USoc 6
Table S11: All PRS for NCDS – childhood and adulthood 8
Table S12: All PRS for NCDS – childhood vs adulthood analysis 14
Table S13: Multiple-testing Results 16
References 31

Table S9: All PRS results for MCS

Environment	Threshold z-scored	SCZ			SCZ Sensitivity			SCZ Wald chi-squared		MDD		
		Beta	95%CI	P-Value	Beta	95%CI	P-Value	chi2	p-value	Beta	95%CI	P-Value
Child SES-by-time	0.01	0.01	0.00-0.01	3.59E-02*						0	-0.00-0.01	1.65E-01
	0.1	0.01	0.00-0.01	2.61E-02*						0.01	0.00-0.01	2.61E-02*
	0.2	0.01	0.00-0.01	2.17E-02*						0.01	-0.00-0.01	5.25E-02
	0.3	0.01	0.00-0.01	3.18E-02*						0.01	0.00-0.01	4.95E-02*
	0.4	0.01	0.00-0.01	3.29E-02*						0.01	-0.00-0.01	5.30E-02
	0.5	0.01	0.00-0.01	3.40E-02*						0.01	0.00-0.01	4.09E-02*
	1	0.01	0.00-0.01	3.32E-02*						0.01	0.00-0.01	3.84E-02*
Child Finance Issues-by-time	0.01	-0.01	-0.06-0.05	8.49E-01						-0.03	-0.09-0.02	2.40E-01
	0.1	-0.01	-0.06-0.04	7.61E-01						-0.02	-0.07-0.03	3.74E-01
	0.2	0	-0.05-0.05	8.50E-01						-0.03	-0.08-0.02	2.84E-01
	0.3	-0.01	-0.05-0.04	8.43E-01						-0.03	-0.08-0.02	3.02E-01
	0.4	-0.01	-0.06-0.04	8.05E-01						-0.03	-0.08-0.02	2.65E-01
	0.5	-0.01	-0.06-0.04	7.81E-01						-0.03	-0.08-0.02	2.60E-01
	1	-0.01	-0.06-0.04	7.96E-01						-0.03	-0.08-0.02	2.82E-01
Child Number of Rooms-by-time	0.01	-0.01	-0.02-0.00	6.06E-02						0	-0.01-0.01	9.60E-01
	0.1	-0.01	-0.01-0.00	1.53E-01						0	-0.01-0.01	9.81E-01
	0.2	-0.01	-0.01-0.00	1.93E-01						0	-0.01-0.01	9.11E-01
	0.3	0	-0.01-0.00	2.71E-01						0	-0.01-0.01	8.25E-01
	0.4	0	-0.01-0.00	2.98E-01						0	-0.01-0.01	7.78E-01
	0.5	0	-0.01-0.00	3.17E-01						0	-0.01-0.01	7.58E-01

	1	0	-0.01-0.00	3.50E-01						0	-0.01-0.01	6.65E-01
**Child Tenure-by-time**	0.01	-0.12	-0.17--0.07	**1.61E-06****	-0.07	-0.12--0.02	5.58E-03	0.95	3.31E-01	-0.03	-0.08-0.02	2.12E-01
	0.1	-0.11	-0.16--0.06	**2.67E-05****	-0.08	-0.14--0.03	2.83E-03	1.44	2.30E-01	-0.01	-0.06-0.03	5.60E-01
	0.2	-0.1	-0.16--0.05	**5.50E-05****	-0.08	-0.14--0.03	4.46E-03	1.4	2.36E-01	0.01	-0.04-0.06	6.70E-01
	0.3	-0.1	-0.15--0.05	**1.29E-04****	-0.08	-0.13--0.02	6.64E-03	1.83	1.76E-01	0.01	-0.04-0.06	6.58E-01
	0.4	-0.09	-0.15--0.04	**2.34E-04****	-0.08	-0.13--0.02	6.26E-03	1.99	1.59E-01	0.01	-0.03-0.06	6.21E-01
	0.5	-0.09	-0.14--0.04	**2.83E-04****	-0.08	-0.13--0.02	7.47E-03	1.95	1.63E-01	0.01	-0.03-0.06	5.90E-01
	1	-0.09	-0.14--0.04	**3.95E-04****	-0.08	-0.13--0.02	8.34E-03	1.47	2.26E-01	0.02	-0.03-0.06	5.14E-01
**Mother taking child for walks-by-time**	0.01	-0.03	-0.10-0.03	3.23E-01						0.03	-0.04-0.10	3.69E-01
	0.1	-0.03	-0.10-0.03	3.28E-01						0.01	-0.06-0.08	8.23E-01
	0.2	-0.04	-0.10-0.03	2.93E-01						0.01	-0.06-0.08	8.02E-01
	0.3	-0.04	-0.10-0.03	2.97E-01						0.01	-0.06-0.08	7.33E-01
	0.4	-0.03	-0.10-0.03	3.23E-01						0.01	-0.06-0.07	8.69E-01
	0.5	-0.03	-0.10-0.03	3.33E-01						0	-0.07-0.07	9.86E-01
	1	-0.03	-0.10-0.03	3.29E-01						0	-0.07-0.07	9.45E-01
**Father taking child for walks-by-time**	0.01	-0.04	-0.14-0.05	3.66E-01						0.02	-0.08-0.11	7.29E-01
	0.1	-0.05	-0.15-0.05	3.27E-01						-0.01	-0.12-0.10	8.27E-01
	0.2	-0.05	-0.16-0.05	3.19E-01						0	-0.11-0.12	9.37E-01
	0.3	-0.05	-0.16-0.05	3.21E-01						0	-0.11-0.12	9.35E-01
	0.4	-0.05	-0.16-0.05	3.31E-01						0.01	-0.11-0.12	9.06E-01
	0.5	-0.05	-0.15-0.06	3.73E-01						0.01	-0.11-0.12	9.04E-01
	1	-0.05	-0.16-0.06	3.58E-01						0	-0.11-0.11	9.65E-01
**Smoking Mother-by-time**	0.01	0.02	-0.03-0.06	4.94E-01						0.01	-0.03-0.05	6.32E-01
	0.1	0.01	-0.04-0.05	8.04E-01						0.01	-0.04-0.05	7.55E-01
	0.2	0.01	-0.04-0.05	7.09E-01						0.01	-0.04-0.05	7.95E-01

	0.3	0.01	-0.04-0.05	6.97E-01			0.01	-0.04-0.05	7.94E-01
	0.4	0.01	-0.04-0.06	6.86E-01			0.01	-0.04-0.05	7.86E-01
	0.5	0.01	-0.04-0.05	7.14E-01			0.01	-0.04-0.05	7.55E-01
	1	0.01	-0.04-0.05	7.36E-01			0.01	-0.04-0.05	7.90E-01
**Parental Marital status-by-time**	0.01	0.02	-0.01-0.04	2.44E-01			-0.01	-0.03-0.02	5.27E-01
	0.1	0.01	-0.01-0.04	3.91E-01			-0.01	-0.03-0.02	6.08E-01
	0.2	0.01	-0.02-0.04	4.50E-01			-0.01	-0.04-0.02	4.45E-01
	0.3	0.01	-0.02-0.04	4.21E-01			-0.01	-0.04-0.02	4.81E-01
	0.4	0.01	-0.02-0.04	4.43E-01			-0.01	-0.04-0.02	4.69E-01
	0.5	0.01	-0.02-0.04	4.31E-01			-0.01	-0.04-0.02	5.46E-01
	1	0.01	-0.02-0.04	4.31E-01			-0.01	-0.03-0.02	6.18E-01
**Alcohol Consumption Mother-by-time**	0.01	0	-0.04-0.03	8.12E-01			-0.02	-0.05-0.01	2.84E-01
	0.1	0	-0.04-0.03	8.39E-01			-0.01	-0.05-0.03	5.94E-01
	0.2	0	-0.04-0.03	8.23E-01			-0.01	-0.06-0.03	5.43E-01
	0.3	0	-0.04-0.03	8.05E-01			-0.01	-0.05-0.03	6.36E-01
	0.4	-0.01	-0.04-0.03	7.75E-01			-0.01	-0.05-0.03	6.21E-01
	0.5	-0.01	-0.04-0.03	7.72E-01			-0.01	-0.05-0.03	6.19E-01
	1	-0.01	-0.04-0.03	7.23E-01			-0.01	-0.05-0.03	6.33E-01
**Alcohol Consumption Father-by-time**	0.01	0.03	-0.05-0.10	4.82E-01			-0.02	-0.09-0.05	5.29E-01
	0.1	0	-0.08-0.08	9.73E-01			-0.04	-0.13-0.05	4.17E-01
	0.2	-0.01	-0.10-0.07	7.66E-01			-0.05	-0.15-0.04	2.85E-01
	0.3	-0.02	-0.11-0.07	6.19E-01			-0.06	-0.16-0.04	2.19E-01
	0.4	-0.03	-0.11-0.06	5.53E-01			-0.05	-0.15-0.04	2.81E-01
	0.5	-0.02	-0.11-0.06	6.07E-01			-0.06	-0.15-0.04	2.64E-01
	1	-0.02	-0.11-0.07	6.12E-01			-0.06	-0.16-0.04	2.17E-01

**Mother reading to child-by-time**	0.01	-0.02	-0.11-0.08	7.15E-01			-0.05	-0.14-0.05	3.12E-01
	0.1	-0.03	-0.13-0.07	5.41E-01			-0.05	-0.14-0.04	2.70E-01
	0.2	-0.03	-0.13-0.06	4.73E-01			-0.04	-0.13-0.06	4.49E-01
	0.3	-0.03	-0.13-0.06	4.96E-01			-0.04	-0.13-0.05	4.24E-01
	0.4	-0.04	-0.13-0.06	4.56E-01			-0.04	-0.13-0.05	4.22E-01
	0.5	-0.04	-0.13-0.06	4.37E-01			-0.04	-0.13-0.05	3.50E-01
	1	-0.04	-0.13-0.06	4.23E-01	-	-	-0.05	-0.13-0.04	3.23E-01
**Father reading to child-by-time**	0.01	-0.09	-0.19-0.01	8.25E-02			-0.04	-0.14-0.06	4.70E-01
	0.1	-0.06	-0.18-0.05	2.83E-01			0.03	-0.09-0.15	6.39E-01
	0.2	-0.06	-0.18-0.05	2.83E-01			0.04	-0.08-0.16	5.42E-01
	0.3	-0.07	-0.19-0.04	2.26E-01			0.05	-0.07-0.17	4.34E-01
	0.4	-0.07	-0.19-0.05	2.42E-01			0.05	-0.07-0.18	3.96E-01
	0.5	-0.07	-0.19-0.04	2.15E-01			0.06	-0.07-0.18	3.76E-01
	1	-0.08	-0.19-0.04	2.13E-01			0.04	-0.08-0.16	5.01E-01

Note: All results were corrected for multiple testing using the Benjamini-Hochberg correction adjusted α = (rank of p-value/number of tests for each threshold) - *α [adjusted α=(rank/560)*0.05]. * = significant, ** = significant after multiple testing. Sensitivity analysis was performed for all statistically significant results after multiple testing only. Beta = beta coefficient, CI = Confidence Interval, All regressions were calculated using STATA v12.1 (1).

Table S10: All PRS results for USoc

Environment	Threshold z-scored	SCZ			MDD			MDD Sensitivity			MDD Wald chi-squared	
		**Beta**	**95%CI**	**P-Value**	**Beta**	**95%CI**	**P-Value**	**Beta**	**95%CI**	**P-Value**	**chi2**	**p-value**
**Adult SES-by-time**	0.01	0	-0.00-0.01	6.63E-01	-0.01	-0.01-0.00	6.60E-02	0	-0.01-0	8.24E-02	1.75	1.86E-01
	0.1	0	-0.00-0.01	8.22E-01	-0.01	-0.02--0.01	4.27E-05	-0.01	-0.01-0	2.05E-03**	0.31	5.80E-01
	0.2	0	-0.00-0.01	3.90E-01	-0.01	-0.02--0.00	4.28E-04	-0.01	-0.01-0	1.67E-02**	0.25	6.15E-01
	0.3	0	-0.00-0.01	3.62E-01	-0.01	-0.01--0.00	9.37E-04	-0.01	-0.01-0	2.89E-02**	0.27	6.06E-01
	0.4	0	-0.00-0.01	3.77E-01	-0.01	-0.01--0.00	6.05E-04	-0.01	-0.01-0	1.66E-02**	0.12	7.33E-01
	0.5	0	-0.00-0.01	4.15E-01	-0.01	-0.01--0.00	1.25E-03	-0.01	-0.01-0	2.36E-02**	0.08	7.71E-01
	1	0	-0.00-0.01	4.10E-01	-0.01	-0.01--0.00	1.10E-03	-0.01	-0.01-0	1.98E-02**	0.06	8.08E-01
**Adult Number of Rooms-by-time**	0.01	0.01	0.00-0.01	**5.21E-04****	0	-0.01--0.00	3.54E-02*					
	0.1	0	0.00-0.01	1.14E-02*	0	-0.01--0.00	1.10E-02*					
	0.2	0	0.00-0.01	1.33E-02*	0	-0.01-0.00	6.01E-02					
	0.3	0	0.00-0.01	3.51E-02*	0	-0.01-0.00	5.92E-02					
	0.4	0	0.00-0.01	4.60E-02*	0	-0.01--0.00	3.98E-02*					
	0.5	0	0.00-0.01	4.32E-02*	0	-0.01-0.00	5.28E-02					
	1	0	-0.00-0.01	7.46E-02	0	-0.01-0.00	5.52E-02					
**Adult Tenure-by-time**	0.01	0.06	-0.01-0.14	7.29E-02	0.16	0.08-0.23	**6.77E-05****	0.14	0.07-0.22	3.14E-04	8.46	3.60E-03
	0.1	0.06	-0.01-0.13	9.10E-02	0.13	0.06-0.20	**3.70E-04****	0.15	0.07-0.22	8.51E-05	10.76	1.00E-03
	0.2	0.05	-0.02-0.12	1.66E-01	0.14	0.07-0.21	**1.20E-04****	0.16	0.08-0.23	3.39E-05	0.65	4.21E-01
	0.3	0.05	-0.02-0.12	1.43E-01	0.14	0.07-0.21	**1.79E-04****	0.15	0.08-0.23	4.24E-05	1.29	2.56E-01
	0.4	0.05	-0.03-0.12	2.09E-01	0.14	0.07-0.21	**1.38E-04****	0.16	0.09-0.24	2.13E-05	8.1	4.40E-03

	0.5	0.04	-0.03-0.11	2.31E-01	0.14	0.07-0.22	**1.20E-04****	0.16	0.09-0.24	2.04E-05	7.64	5.70E-03
	1	0.05	-0.02-0.12	1.55E-01	0.14	0.07-0.21	**1.63E-04****	0.16	0.09-0.23	2.49E-05	1.15	2.84E-01
**Adult Employment-by-time**	0.01	-0.02	-0.07-0.03	4.59E-01	0.01	-0.04-0.06	7.88E-01					
	0.1	-0.02	-0.07-0.03	5.01E-01	0.01	-0.04-0.06	6.84E-01					
	0.2	-0.01	-0.06-0.04	6.87E-01	0.02	-0.03-0.07	3.45E-01					
	0.3	-0.01	-0.06-0.04	7.46E-01	0.02	-0.03-0.07	3.96E-01					
	0.4	-0.02	-0.07-0.03	5.30E-01	0.02	-0.03-0.06	5.22E-01					
	0.5	-0.02	-0.07-0.03	4.02E-01	0.02	-0.03-0.06	5.31E-01					
	1	-0.02	-0.07-0.03	4.21E-01	0.02	-0.03-0.07	4.75E-01					
**Adult Finance Issues-by-time**	0.01	0.01	-0.04-0.05	6.76E-01	-0.02	-0.06-0.03	4.43E-01					
	0.1	-0.02	-0.06-0.03	4.92E-01	-0.01	-0.05-0.04	7.80E-01					
	0.2	-0.01	-0.06-0.03	5.46E-01	-0.01	-0.06-0.03	5.77E-01					
	0.3	-0.01	-0.06-0.03	6.04E-01	-0.02	-0.06-0.02	3.78E-01					
	0.4	-0.02	-0.06-0.03	4.87E-01	-0.03	-0.07-0.02	2.03E-01					
	0.5	-0.01	-0.06-0.03	5.63E-01	-0.03	-0.07-0.02	2.15E-01					
	1	-0.02	-0.06-0.03	4.68E-01	-0.03	-0.07-0.02	2.10E-01					
**Adult Income-by-time**	0.01	0	-0.00-0.01	7.64E-01	0	-0.01-0.00	3.30E-01					
	0.1	0	-0.00-0.01	4.72E-01	0	-0.01-0.00	6.21E-01					
	0.2	0	-0.00-0.01	3.70E-01	0	-0.01-0.00	2.05E-01					
	0.3	0	-0.00-0.01	5.24E-01	0	-0.01-0.00	1.80E-01					
	0.4	0	-0.00-0.01	6.22E-01	0	-0.01-0.00	2.81E-01					
	0.5	0	-0.00-0.01	6.55E-01	0	-0.01-0.00	3.34E-01					
	1	0	-0.00-0.01	5.71E-01	0	-0.01-0.00	3.88E-01					

Note: All results were corrected for multiple testing using the Benjamini-Hochberg correction adjusted α = (rank of p-value/number of tests for each threshold) - *α [adjusted α=(rank/560)*0.05]. * = significant, ** = significant after multiple testing. Sensitivity analysis was performed for all statistically significant results for MDD only (as no SCZ diagnosis is available in USoc) after multiple testing. Beta = beta coefficient, CI = Confidence Interval, All regressions were calculated using STATA v12.1 (1).

Table S11: All PRS for NCDS – childhood and adulthood

Environment	Threshold z-scored	SCZ			MDD		
		Beta	95%CI	P-Value	Beta	95%CI	P-Value
**Childhood**							
**Child SES-by-time**	0.01	0	-0.01-0.01	6.42E-01	0.01	-0.00-0.01	2.75E-01
	0.1	0	-0.01-0.01	4.82E-01	0.01	-0.00-0.02	1.76E-01
	0.2	0	-0.01-0.01	6.62E-01	0.01	-0.00-0.01	2.80E-01
	0.3	0	-0.01-0.01	5.19E-01	0.01	-0.00-0.02	2.51E-01
	0.4	0	-0.01-0.01	5.98E-01	0.01	-0.00-0.01	2.69E-01
	0.5	0	-0.01-0.01	5.82E-01	0	-0.00-0.01	3.22E-01
	1	0	-0.01-0.01	5.32E-01	0.01	-0.00-0.01	2.56E-01
**Child Finance Issues-by-time**	0.01	-0.06	-0.14-0.02	1.42E-01	0	-0.08-0.08	9.67E-01
	0.1	-0.05	-0.13-0.02	1.71E-01	-0.01	-0.09-0.06	7.07E-01
	0.2	-0.05	-0.13-0.03	1.90E-01	0.01	-0.07-0.09	7.67E-01
	0.3	-0.05	-0.13-0.03	2.28E-01	0.01	-0.07-0.09	8.36E-01
	0.4	-0.05	-0.13-0.03	2.32E-01	0	-0.08-0.08	9.85E-01
	0.5	-0.05	-0.12-0.03	2.55E-01	0	-0.08-0.08	9.78E-01
	1	-0.05	-0.12-0.03	2.54E-01	0	-0.08-0.08	9.43E-01
**Child Number of Rooms-by-time**	0.01	0.01	-0.00-0.02	2.42E-01	0	-0.01-0.01	9.44E-01
	0.1	0.01	-0.00-0.02	6.89E-02	0	-0.01-0.01	5.24E-01
	0.2	0.01	-0.00-0.02	5.65E-02	0.01	-0.00-0.02	2.54E-01
	0.3	0.01	-0.00-0.02	5.41E-02	0.01	-0.01-0.02	3.07E-01
	0.4	0.01	-0.00-0.02	7.34E-02	0.01	-0.00-0.02	2.62E-01

	0.5	0.01	-0.00-0.02	7.55E-02	0.01	-0.00-0.02	1.89E-01
	1	0.01	0.00-0.02	3.93E-02*	0.01	-0.00-0.02	1.87E-01
**Child Tenure-by-time**	0.01	0.01	-0.07-0.09	8.07E-01	0.03	-0.05-0.12	4.16E-01
	0.1	0.03	-0.05-0.12	4.17E-01	0.03	-0.05-0.11	4.28E-01
	0.2	0.03	-0.05-0.11	4.54E-01	0.06	-0.02-0.14	1.41E-01
	0.3	0.04	-0.04-0.12	3.51E-01	0.04	-0.04-0.12	3.08E-01
	0.4	0.03	-0.05-0.12	4.27E-01	0.05	-0.04-0.13	2.70E-01
	0.5	0.04	-0.05-0.12	3.93E-01	0.04	-0.04-0.12	3.03E-01
	1	0.04	-0.05-0.12	3.75E-01	0.05	-0.04-0.13	2.73E-01
**Mother taking child for walks-by-time**	0.01	0.02	-0.14-0.19	7.71E-01	0.07	-0.10-0.23	4.09E-01
	0.1	0.01	-0.15-0.18	8.76E-01	0.02	-0.15-0.18	8.43E-01
	0.2	0.01	-0.15-0.18	8.80E-01	0	-0.16-0.17	9.71E-01
	0.3	0	-0.16-0.17	9.58E-01	-0.03	-0.20-0.13	6.93E-01
	0.4	0.01	-0.15-0.18	8.77E-01	-0.04	-0.21-0.13	6.33E-01
	0.5	0.01	-0.15-0.18	8.96E-01	-0.04	-0.20-0.13	6.63E-01
	1	-0.01	-0.17-0.16	9.18E-01	-0.03	-0.20-0.14	7.17E-01
**Father taking child for walks-by-time**	0.01	-0.05	-0.15-0.05	3.59E-01	0	-0.10-0.10	9.31E-01
	0.1	0.04	-0.06-0.14	4.79E-01	-0.03	-0.13-0.07	6.16E-01
	0.2	0.04	-0.06-0.14	4.16E-01	-0.02	-0.12-0.08	7.14E-01
	0.3	0.04	-0.06-0.14	4.49E-01	-0.04	-0.14-0.06	4.45E-01
	0.4	0.04	-0.06-0.14	4.15E-01	-0.03	-0.13-0.07	4.95E-01
	0.5	0.04	-0.06-0.14	4.51E-01	-0.04	-0.14-0.06	4.51E-01
	1	0.04	-0.06-0.14	4.73E-01	-0.03	-0.13-0.07	5.11E-01
	0.01	0	-0.06-0.07	9.09E-01	0.02	-0.04-0.09	4.52E-01

**Mother interest in child education-by-time**	0.1	0.02	-0.05-0.08	6.12E-01	0.03	-0.03-0.09	3.45E-01
	0.2	0.01	-0.06-0.07	8.19E-01	0.04	-0.03-0.10	2.63E-01
	0.3	0.01	-0.05-0.08	7.06E-01	0.04	-0.02-0.11	1.73E-01
	0.4	0.01	-0.05-0.08	6.72E-01	0.04	-0.02-0.10	2.16E-01
	0.5	0.01	-0.06-0.07	8.17E-01	0.04	-0.02-0.10	2.14E-01
	1	0	-0.06-0.07	9.01E-01	0.04	-0.02-0.10	2.13E-01
**Father involvement in childcare-by-time**	0.01	-0.01	-0.09-0.08	8.75E-01	-0.01	-0.10-0.07	7.75E-01
	0.1	-0.02	-0.10-0.07	6.78E-01	0.03	-0.05-0.11	4.86E-01
	0.2	-0.03	-0.11-0.06	5.50E-01	0.04	-0.05-0.12	3.75E-01
	0.3	-0.02	-0.11-0.06	6.20E-01	0.04	-0.05-0.12	3.84E-01
	0.4	-0.01	-0.09-0.07	8.01E-01	0.04	-0.05-0.12	3.69E-01
	0.5	-0.01	-0.09-0.08	8.73E-01	0.04	-0.04-0.13	3.10E-01
	1	-0.01	-0.09-0.08	8.75E-01	0.04	-0.04-0.13	3.22E-01
**Father interest in child education-by-time**	0.01	0.06	-0.01-0.13	1.13E-01	0.02	-0.05-0.09	6.32E-01
	0.1	0.06	-0.01-0.13	1.06E-01	0.04	-0.03-0.11	3.07E-01
	0.2	0.04	-0.03-0.11	2.42E-01	0.04	-0.03-0.11	2.96E-01
	0.3	0.04	-0.03-0.11	2.29E-01	0.04	-0.03-0.11	2.83E-01
	0.4	0.04	-0.03-0.11	2.61E-01	0.03	-0.04-0.11	3.31E-01
	0.5	0.04	-0.03-0.11	3.12E-01	0.03	-0.04-0.10	3.79E-01
	1	0.04	-0.03-0.11	2.87E-01	0.03	-0.04-0.10	3.53E-01
**Father employment-by-time**	0.01	0.02	-0.13-0.17	7.74E-01	0.03	-0.12-0.19	6.64E-01
	0.1	-0.02	-0.17-0.13	7.69E-01	0.04	-0.08-0.16	5.28E-01
	0.2	-0.01	-0.16-0.15	9.47E-01	-0.02	-0.18-0.14	8.07E-01
	0.3	0.03	-0.12-0.18	7.05E-01	0	-0.16-0.16	9.81E-01
	0.4	0.03	-0.12-0.18	7.12E-01	-0.03	-0.18-0.13	7.53E-01

	0.5	0.03	-0.12-0.18	6.82E-01	-0.03	-0.18-0.13	7.47E-01
	1	0.03	-0.13-0.18	7.30E-01	-0.01	-0.17-0.14	8.55E-01
**Adulthood**							
**Adult SES-by-time**	0.01	0.01	0.00-0.02	4.69E-02*	0	-0.01-0.01	6.27E-01
	0.1	0.01	0.00-0.02	2.94E-02*	0	-0.01-0.01	8.27E-01
	0.2	0.01	-0.00-0.02	6.87E-02	0	-0.01-0.01	5.33E-01
	0.3	0.01	-0.00-0.02	6.01E-02	0	-0.01-0.01	4.24E-01
	0.4	0.01	-0.00-0.02	1.04E-01	0	-0.01-0.01	4.07E-01
	0.5	0.01	-0.00-0.02	8.55E-02	0	-0.01-0.01	3.63E-01
	1	0.01	-0.00-0.02	8.67E-02	0	-0.01-0.01	4.44E-01
**Adult Number of Rooms-by-time**	0.01	-0.01	-0.02-0.00	2.62E-01	0	-0.01-0.01	7.99E-01
	0.1	-0.01	-0.02-0.01	3.61E-01	-0.01	-0.02-0.00	2.02E-01
	0.2	-0.01	-0.02-0.00	1.62E-01	-0.01	-0.02-0.01	2.87E-01
	0.3	-0.01	-0.02-0.00	1.20E-01	-0.01	-0.02-0.01	2.93E-01
	0.4	-0.01	-0.02-0.00	1.84E-01	0	-0.02-0.01	4.53E-01
	0.5	-0.01	-0.02-0.00	1.57E-01	0	-0.01-0.01	5.32E-01
	1	-0.01	-0.02-0.00	1.39E-01	0	-0.01-0.01	5.55E-01
**Adult Tenure-by-time**	0.01	-0.02	-0.08-0.03	4.39E-01	0.03	-0.03-0.09	2.79E-01
	0.1	-0.06	-0.12--0.00	4.39E-02*	0	-0.05-0.06	9.38E-01
	0.2	-0.06	-0.11-0.00	5.65E-02	-0.01	-0.07-0.04	6.12E-01
	0.3	-0.06	-0.12--0.00	4.68E-02*	-0.01	-0.07-0.04	6.29E-01
	0.4	-0.06	-0.11--0.00	4.85E-02*	-0.01	-0.07-0.04	6.79E-01

	0.5	-0.06	-0.12--0.00	4.01E-02*	-0.01	-0.07-0.04	6.39E-01
	1	-0.06	-0.12--0.00	3.97E-02*	-0.02	-0.07-0.04	5.67E-01
**Adult Employment-by-time**	0.01	0.07	-0.04-0.17	2.01E-01	0.07	-0.03-0.17	1.57E-01
	0.1	0.06	-0.03-0.16	2.03E-01	0.04	-0.05-0.14	3.78E-01
	0.2	0.07	-0.03-0.16	1.91E-01	0.04	-0.05-0.14	3.88E-01
	0.3	0.08	-0.02-0.18	1.22E-01	0.04	-0.05-0.14	3.70E-01
	0.4	0.07	-0.02-0.17	1.39E-01	0.05	-0.05-0.14	3.37E-01
	0.5	0.08	-0.02-0.18	1.23E-01	0.04	-0.05-0.14	3.78E-01
	1	0.07	-0.03-0.17	1.80E-01	0.04	-0.06-0.13	4.67E-01
**Adult Marital status-by-time**	0.01	0.04	0.01-0.08	2.20E-02*	0.02	-0.01-0.06	1.62E-01
	0.1	0.02	-0.02-0.05	3.60E-01	0.05	0.02-0.09	4.71E-03*
	0.2	0.02	-0.01-0.06	2.50E-01	0.04	0.01-0.08	2.28E-02*
	0.3	0.02	-0.02-0.05	3.32E-01	0.04	0.00-0.07	3.60E-02*
	0.4	0.02	-0.02-0.05	3.45E-01	0.04	0.01-0.08	2.19E-02*
	0.5	0.02	-0.02-0.05	3.75E-01	0.04	0.00-0.07	3.67E-02*
	1	0.02	-0.02-0.05	3.73E-01	0.04	0.00-0.07	4.30E-02*
**Adult Smoking-by-time**	0.01	-0.01	-0.08-0.07	8.90E-01	0.07	-0.01-0.14	7.59E-02
	0.1	0.02	-0.05-0.10	5.25E-01	0.05	-0.03-0.12	2.25E-01
	0.2	0.01	-0.06-0.09	7.19E-01	0.06	-0.02-0.13	1.45E-01
	0.3	0.02	-0.05-0.10	5.40E-01	0.06	-0.02-0.13	1.48E-01
	0.4	0.02	-0.06-0.09	6.33E-01	0.05	-0.02-0.13	1.68E-01

	0.5	0.01	-0.06-0.09	7.07E-01	0.06	-0.02-0.13	1.57E-01
	1	0.01	-0.06-0.09	7.16E-01	0.06	-0.02-0.14	1.29E-01

Note: All results were corrected for multiple testing using the Benjamini-Hochberg correction adjusted α=(rank of p-value/number of tests for each threshold) - *α [adjusted α=(rank/560)*0.05]. * = significant, ** = significant after multiple testing. Sensitivity analysis was performed for all statistically significant results after multiple testing only. Beta = beta coefficient, CI = Confidence Interval, All regressions were calculated using STATA v12.1 (1).

Table S12: All PRS for NCDS – childhood vs adulthood analysis

Environment	Threshold	SCZ			SCZ Sensitivity			SCZ Wald chi-squared		MDD			MDD Sensitivity			MDD Wald chi-squared	
		Beta	95%CI	P-Value	Beta	95%CI	P-Value	chi2	p-value	Beta	95%CI	P-Value	Beta	95%CI	P-Value	chi2	p-value
Family SES vs adult SES (0=child, 1=adult)	0.01	0	-0.02-0.02	9.54E-01	0	-0.04	8.82E-01	0.15	7.00E-01	0.04	0.02-0.05	**7.12E-06****	0.04	0.02-0.06	2.31E-06	0.16	6.90E-01
	0.1	0.02	0.00-0.03	1.88E-02*	0.02	0.01-0.04	8.79E-03	0.75	3.88E-01	0.03	0.02-0.05	**6.72E-05****	0.04	0.02-0.06	4.66E-06	1.29	2.56E-01
	0.2	0.02	0.01-0.04	4.97E-03*	0.03	0.01-0.04	1.77E-03	1.71	1.91E-01	0.04	0.02-0.05	**4.10E-06****	0.05	0.03-0.06	4.86E-07	2.43	1.19E-01
	0.3	0.02	0.01-0.04	**2.27E-03****	0.03	0.01-0.05	8.02E-04	1.28	2.59E-01	0.04	0.02-0.05	**1.04E-06****	0.05	0.03-0.07	5.18E-08	3.1	7.81E-02
	0.4	0.03	0.01-0.04	**1.11E-03****	0.03	0.01-0.05	5.44E-04	1.08	2.98E-01	0.04	0.02-0.05	**3.28E-06****	0.05	0.03-0.06	1.59E-07	2.73	9.86E-02
	0.5	0.02	0.01-0.04	**1.96E-03****	0.03	0.01-0.05	8.77E-04	1.17	2.80E-01	0.04	0.02-0.05	**2.00E-06****	0.05	0.03-0.07	5.22E-08	2.69	1.01E-01
	1	0.02	0.01-0.04	**1.75E-03****	0.03	0.01-0.05	7.05E-04	1.19	2.76E-01	0.04	0.02-0.05	**3.12E-06****	0.05	0.03-0.07	5.94E-08	3.06	8.01E-02
Father's employment vs adult employment (0=child, 1=adult)	0.01	0.05	-0.08-0.17	4.43E-01						0.07	-0.06-0.20	2.76E-01					
	0.1	-0.02	-0.15-0.10	7.24E-01						-0.18	-0.31--0.05	5.48E-03*					
	0.2	-0.03	-0.16-0.09	6.00E-01						-0.17	-0.30--0.05	7.08E-03*					
	0.3	-0.02	-0.15-0.10	7.08E-01						-0.16	-0.28--0.03	1.60E-02*					
	0.4	-0.02	-0.14-0.11	8.09E-01						-0.17	-0.29--0.04	1.07E-02*					
	0.5	-0.02	-0.14-0.11	7.90E-01						-0.16	-0.29--0.04	1.19E-02*					
	1	-0.02	-0.15-0.11	7.48E-01						-0.18	-0.31--0.05	6.29E-03*					
Family number of rooms vs adult number of rooms (0=child, 1=adult)	0.01	-0.01	-0.03-0.00	1.10E-01						0.02	-0.00-0.03	6.81E-02					
	0.1	0	-0.02-0.02	9.06E-01						0.01	-0.01-0.03	2.16E-01					
	0.2	-0.01	-0.03-0.01	4.51E-01						0.01	-0.00-0.03	1.46E-01					
	0.3	-0.01	-0.03-0.01	2.52E-01						0.02	-0.00-0.03	1.01E-01					
	0.4	-0.01	-0.03-0.01	3.24E-01						0.01	-0.00-0.03	1.28E-01					
	0.5	-0.01	-0.03-0.01	3.42E-01						0.01	-0.00-0.03	1.29E-01					

	1	-0.01	-0.03-0.01	3.95E-01			0.01	-0.01-0.03	1.65E-01					
**Family tenure vs adult tenure (0=child, 1=adult)**	0.01	0	-0.07-0.07	9.77E-01			-0.14	-0.21--0.07	**1.11E-04****	-0.2	-0.28--0.11	3.76E-06	0.25	6.15E-01
	0.1	-0.04	-0.11-0.03	2.65E-01			-0.07	-0.14-0.00	5.15E-02	-0.15	-0.24--0.07	3.22E-04	0.33	5.65E-01
	0.2	-0.04	-0.11-0.03	2.74E-01			-0.06	-0.13-0.01	8.45E-02	-0.14	-0.22--0.05	1.18E-03	1.08	2.98E-01
	0.3	-0.05	-0.12-0.02	1.25E-01			-0.06	-0.13-0.01	1.08E-01	-0.14	-0.22--0.05	1.29E-03	1.5	2.21E-01
	0.4	-0.05	-0.12-0.02	1.30E-01			-0.05	-0.12-0.02	1.43E-01	-0.12	-0.20--0.04	3.96E-03	1.28	2.58E-01
	0.5	-0.06	-0.13-0.01	1.09E-01			-0.06	-0.13-0.01	1.14E-01	-0.13	-0.22--0.05	1.55E-03	1.08	2.98E-01
	1	-0.05	-0.12-0.02	1.43E-01			-0.05	-0.12-0.02	1.48E-01	-0.13	-0.21--0.05	1.71E-03	1.43	2.31E-01
**Parental marital status vs adult marital status (0=child, 1=adult)**	0.01	0.06	-0.12-0.24	5.19E-01			-0.06	-0.24-0.12	5.25E-01					
	0.1	0.1	-0.07-0.28	2.52E-01			0.01	-0.18-0.19	9.45E-01					
	0.2	0.1	-0.08-0.27	2.97E-01			0	-0.18-0.18	9.99E-01					
	0.3	0.11	-0.07-0.28	2.49E-01			-0.02	-0.21-0.16	7.94E-01					
	0.4	0.12	-0.06-0.30	1.84E-01			-0.05	-0.23-0.14	6.28E-01					
	0.5	0.12	-0.06-0.30	1.75E-01			-0.05	-0.24-0.13	5.62E-01					
	1	0.13	-0.05-0.31	1.49E-01			-0.04	-0.23-0.14	6.35E-01					
**Maternal smoking prior and during pregnancy vs adult smoking (0=child, 1=adult)**	0.01	-0.05	-0.13-0.03	2.27E-01			0.05	-0.03-0.13	2.35E-01					
	0.1	-0.03	-0.11-0.05	4.68E-01			0.04	-0.05-0.12	3.89E-01					
	0.2	-0.01	-0.10-0.07	7.61E-01			0.06	-0.02-0.15	1.37E-01					
	0.3	-0.02	-0.10-0.06	6.49E-01			0.07	-0.01-0.15	9.99E-02					
	0.4	-0.02	-0.10-0.06	6.36E-01			0.05	-0.04-0.13	2.73E-01					
	0.5	-0.02	-0.11-0.06	5.79E-01			0.04	-0.04-0.13	2.92E-01					
	1	-0.02	-0.11-0.06	5.98E-01			0.04	-0.04-0.13	3.14E-01					

Note: All results were corrected for multiple testing using the Benjamini-Hochberg correction adjusted α=(rank of p-value/number of tests for each threshold) - *α [adjusted α=(rank/560)*0.05]. * = significant, ** = significant after multiple testing. Sensitivity analysis was performed for all statistically significant results after multiple testing only. Beta = beta coefficient, CI = Confidence Interval, All regressions were calculated using STATA v12.1 (1).

Table S13: Multiple-testing Results

Environment	SCZ or MDD	Cohort	Threshold	Beta	CI	p-value	rank	adj alpha	sig?
SES child vs adult	MDD	NCDS	0.3	0.04	0.02-0.05	1.04E-06	1	8.93E-05	yes
Tenure Child	SCZ	MCS	0.01	-0.12	-0.17--0.07	1.61E-06	2	1.79E-04	yes
SES child vs adult	MDD	NCDS	0.5	0.04	0.02-0.05	2.00E-06	3	2.68E-04	yes
SES child vs adult	MDD	NCDS	1	0.04	0.02-0.05	3.12E-06	4	3.57E-04	yes
SES child vs adult	MDD	NCDS	0.4	0.04	0.02-0.05	3.28E-06	5	4.46E-04	yes
SES child vs adult	MDD	NCDS	0.2	0.04	0.02-0.05	4.10E-06	6	5.36E-04	yes
SES child vs adult	MDD	NCDS	0.01	0.04	0.02-0.05	7.12E-06	7	6.25E-04	yes
Tenure Child	SCZ	MCS	0.1	-0.11	-0.16--0.06	2.67E-05	8	7.14E-04	yes
SES Adult	MDD	USoc	0.1	-0.01	-0.02--0.01	4.27E-05	9	8.04E-04	yes
Tenure Child	SCZ	MCS	0.2	-0.1	-0.16--0.05	5.50E-05	10	8.93E-04	yes
SES child vs adult	MDD	NCDS	0.1	0.03	0.02-0.05	6.72E-05	11	9.82E-04	yes
Tenure Adult	MDD	USoc	0.01	0.16	0.08-0.23	6.77E-05	12	1.07E-03	yes
Tenure child vs adult	MDD	NCDS	0.01	-0.14	-0.21--0.07	1.11E-04	13	1.16E-03	yes
Tenure Adult	MDD	USoc	0.5	0.14	0.07-0.22	1.20E-04	14	1.25E-03	yes
Tenure Adult	MDD	USoc	0.2	0.14	0.07-0.21	1.20E-04	15	1.34E-03	yes
Tenure Child	SCZ	MCS	0.3	-0.1	-0.15--0.05	1.29E-04	16	1.43E-03	yes
Tenure Adult	MDD	USoc	0.4	0.14	0.07-0.21	1.38E-04	17	1.52E-03	yes
Tenure Adult	MDD	USoc	1	0.14	0.07-0.21	1.63E-04	18	1.61E-03	yes
Tenure Adult	MDD	USoc	0.3	0.14	0.07-0.21	1.79E-04	19	1.70E-03	yes
Tenure Child	SCZ	MCS	0.4	-0.09	-0.15--0.04	2.34E-04	20	1.79E-03	yes
Tenure Child	SCZ	MCS	0.5	-0.09	-0.14--0.04	2.83E-04	21	1.88E-03	yes
Tenure Adult	MDD	USoc	0.1	0.13	0.06-0.20	3.70E-04	22	1.96E-03	yes
Tenure Child	SCZ	MCS	1	-0.09	-0.14--0.04	3.95E-04	23	2.05E-03	yes
SES Adult	MDD	USoc	0.2	-0.01	-0.02--0.00	4.28E-04	24	2.14E-03	yes
Number of Rooms Adult	SCZ	USoc	0.01	0.01	0.00-0.01	5.21E-04	25	2.23E-03	yes
SES Adult	MDD	USoc	0.4	-0.01	-0.01--0.00	6.05E-04	26	2.32E-03	yes
SES Adult	MDD	USoc	0.3	-0.01	-0.01--0.00	9.37E-04	27	2.41E-03	yes
SES Adult	MDD	USoc	1	-0.01	-0.01--0.00	1.10E-03	28	2.50E-03	yes
SES child vs adult	SCZ	NCDS	0.4	0.03	0.01-0.04	1.11E-03	29	2.59E-03	yes
SES Adult	MDD	USoc	0.5	-0.01	-0.01--0.00	1.25E-03	30	2.68E-03	yes
SES child vs adult	SCZ	NCDS	1	0.02	0.01-0.04	1.75E-03	31	2.77E-03	yes
SES child vs adult	SCZ	NCDS	0.5	0.02	0.01-0.04	1.96E-03	32	2.86E-03	yes

SES child vs adult	SCZ	NCDS	0.3	0.02	0.01-0.04	2.27E-03	33	2.95E-03	yes
Marital status Adult	MDD	NCDS	0.1	0.05	0.02-0.09	4.71E-03	34	3.04E-03	no
SES child vs adult	SCZ	NCDS	0.2	0.02	0.01-0.04	4.97E-03	35	3.13E-03	no
Employment father vs adult	MDD	NCDS	0.1	-0.18	-0.31--0.05	5.48E-03	36	3.21E-03	no
Employment father vs adult	MDD	NCDS	1	-0.18	-0.31--0.05	6.29E-03	37	3.30E-03	no
Employment father vs adult	MDD	NCDS	0.2	-0.17	-0.30--0.05	7.08E-03	38	3.39E-03	no
Employment father vs adult	MDD	NCDS	0.4	-0.17	-0.29--0.04	1.07E-02	39	3.48E-03	no
Number of Rooms Adult	MDD	USoc	0.1	0	-0.01--0.00	1.10E-02	40	3.57E-03	no
Number of Rooms Adult	SCZ	USoc	0.1	0	0.00-0.01	1.14E-02	41	3.66E-03	no
Employment father vs adult	MDD	NCDS	0.5	-0.16	-0.29--0.04	1.19E-02	42	3.75E-03	no
Number of Rooms Adult	SCZ	USoc	0.2	0	0.00-0.01	1.33E-02	43	3.84E-03	no
Employment father vs adult	MDD	NCDS	0.3	-0.16	-0.28--0.03	1.60E-02	44	3.93E-03	no
SES child vs adult	SCZ	NCDS	0.1	0.02	0.00-0.03	1.88E-02	45	4.02E-03	no
SES Child	SCZ	MCS	0.2	0.01	0.00-0.01	2.17E-02	46	4.11E-03	no
Marital status Adult	MDD	NCDS	0.4	0.04	0.01-0.08	2.19E-02	47	4.20E-03	no
Marital status Adult	SCZ	NCDS	0.01	0.04	0.01-0.08	2.20E-02	48	4.29E-03	no
Marital status Adult	MDD	NCDS	0.2	0.04	0.01-0.08	2.28E-02	49	4.38E-03	no
SES Child	MDD	MCS	0.1	0.01	0.00-0.01	2.61E-02	50	4.46E-03	no
SES Child	SCZ	MCS	0.1	0.01	0.00-0.01	2.61E-02	51	4.55E-03	no
SES Adult	SCZ	NCDS	0.1	0.01	0.00-0.02	2.94E-02	52	4.64E-03	no
SES Child	SCZ	MCS	0.3	0.01	0.00-0.01	3.18E-02	53	4.73E-03	no
SES Child	SCZ	MCS	0.4	0.01	0.00-0.01	3.29E-02	54	4.82E-03	no
SES Child	SCZ	MCS	1	0.01	0.00-0.01	3.32E-02	55	4.91E-03	no
SES Child	SCZ	MCS	0.5	0.01	0.00-0.01	3.40E-02	56	5.00E-03	no
Number of Rooms Adult	SCZ	USoc	0.3	0	0.00-0.01	3.51E-02	57	5.09E-03	no
Number of Rooms Adult	MDD	USoc	0.01	0	-0.01--0.00	3.54E-02	58	5.18E-03	no
SES Child	SCZ	MCS	0.01	0.01	0.00-0.01	3.59E-02	59	5.27E-03	no
Marital status Adult	MDD	NCDS	0.3	0.04	0.00-0.07	3.60E-02	60	5.36E-03	no
Marital status Adult	MDD	NCDS	0.5	0.04	0.00-0.07	3.67E-02	61	5.45E-03	no
SES Child	MDD	MCS	1	0.01	0.00-0.01	3.84E-02	62	5.54E-03	no
Number of Rooms Child	SCZ	NCDS	1	0.01	0.00-0.02	3.93E-02	63	5.63E-03	no
Tenure Adult	SCZ	NCDS	1	-0.06	-0.12--0.00	3.97E-02	64	5.71E-03	no
Number of Rooms Adult	MDD	USoc	0.4	0	-0.01--0.00	3.98E-02	65	5.80E-03	no
Tenure Adult	SCZ	NCDS	0.5	-0.06	-0.12--0.00	4.01E-02	66	5.89E-03	no
SES Child	MDD	MCS	0.5	0.01	0.00-0.01	4.09E-02	67	5.98E-03	no
Marital status Adult	MDD	NCDS	1	0.04	0.00-0.07	4.30E-02	68	6.07E-03	no

Number of Rooms Adult	SCZ	USoc	0.5	0	0.00-0.01	4.32E-02	69	6.16E-03	no
Tenure Adult	SCZ	NCDS	0.1	-0.06	-0.12--0.00	4.39E-02	70	6.25E-03	no
Number of Rooms Adult	SCZ	USoc	0.4	0	0.00-0.01	4.60E-02	71	6.34E-03	no
Tenure Adult	SCZ	NCDS	0.3	-0.06	-0.12--0.00	4.68E-02	72	6.43E-03	no
SES Adult	SCZ	NCDS	0.01	0.01	0.00-0.02	4.69E-02	73	6.52E-03	no
Tenure Adult	SCZ	NCDS	0.4	-0.06	-0.11--0.00	4.85E-02	74	6.61E-03	no
SES Child	MDD	MCS	0.3	0.01	0.00-0.01	4.95E-02	75	6.70E-03	no
Tenure child vs adult	MDD	NCDS	0.1	-0.07	-0.14-0.00	5.15E-02	76	6.79E-03	no
SES Child	MDD	MCS	0.2	0.01	-0.00-0.01	5.25E-02	77	6.88E-03	no
Number of Rooms Adult	MDD	USoc	0.5	0	-0.01-0.00	5.28E-02	78	6.96E-03	no
SES Child	MDD	MCS	0.4	0.01	-0.00-0.01	5.30E-02	79	7.05E-03	no
Number of Rooms Child	SCZ	NCDS	0.3	0.01	-0.00-0.02	5.41E-02	80	7.14E-03	no
Number of Rooms Adult	MDD	USoc	1	0	-0.01-0.00	5.52E-02	81	7.23E-03	no
Number of Rooms Child	SCZ	NCDS	0.2	0.01	-0.00-0.02	5.65E-02	82	7.32E-03	no
Tenure Adult	SCZ	NCDS	0.2	-0.06	-0.11-0.00	5.65E-02	83	7.41E-03	no
Number of Rooms Adult	MDD	USoc	0.3	0	-0.01-0.00	5.92E-02	84	7.50E-03	no
SES Adult	SCZ	NCDS	0.3	0.01	-0.00-0.02	6.01E-02	85	7.59E-03	no
Number of Rooms Adult	MDD	USoc	0.2	0	-0.01-0.00	6.01E-02	86	7.68E-03	no
Number of Rooms Child	SCZ	MCS	0.01	-0.01	-0.02-0.00	6.06E-02	87	7.77E-03	no
SES Adult	MDD	USoc	0.01	-0.01	-0.01-0.00	6.60E-02	88	7.86E-03	no
Number of Rooms child vs adult	MDD	NCDS	0.01	0.02	-0.00-0.03	6.81E-02	89	7.95E-03	no
SES Adult	SCZ	NCDS	0.2	0.01	-0.00-0.02	6.87E-02	90	8.04E-03	no
Number of Rooms Child	SCZ	NCDS	0.1	0.01	-0.00-0.02	6.89E-02	91	8.13E-03	no
Tenure Adult	SCZ	USoc	0.01	0.06	-0.01-0.14	7.29E-02	92	8.21E-03	no
Number of Rooms Child	SCZ	NCDS	0.4	0.01	-0.00-0.02	7.34E-02	93	8.30E-03	no
Number of Rooms Adult	SCZ	USoc	1	0	-0.00-0.01	7.46E-02	94	8.39E-03	no
Number of Rooms Child	SCZ	NCDS	0.5	0.01	-0.00-0.02	7.55E-02	95	8.48E-03	no
Smoking Adult	MDD	NCDS	0.01	0.07	-0.01-0.14	7.59E-02	96	8.57E-03	no
Father Reads to Child	SCZ	MCS	0.01	-0.09	-0.19-0.01	8.25E-02	97	8.66E-03	no
Tenure child vs adult	MDD	NCDS	0.2	-0.06	-0.13-0.01	8.45E-02	98	8.75E-03	no
SES Adult	SCZ	NCDS	0.5	0.01	-0.00-0.02	8.55E-02	99	8.84E-03	no
SES Adult	SCZ	NCDS	1	0.01	-0.00-0.02	8.67E-02	100	8.93E-03	no
Tenure Adult	SCZ	USoc	0.1	0.06	-0.01-0.13	9.10E-02	101	9.02E-03	no
Smoking mother prior & during pregnancy vs adult	MDD	NCDS	0.3	0.07	-0.01-0.15	9.99E-02	102	9.11E-03	no
Number of Rooms child vs adult	MDD	NCDS	0.3	0.02	-0.00-0.03	1.01E-01	103	9.20E-03	no
SES Adult	SCZ	NCDS	0.4	0.01	-0.00-0.02	1.04E-01	104	9.29E-03	no

Father's interest in child's education	SCZ	NCDS	0.1	0.06	-0.01-0.13	1.06E-01	105	9.38E-03	no
Tenure child vs adult	MDD	NCDS	0.3	-0.06	-0.13-0.01	1.08E-01	106	9.46E-03	no
Tenure child vs adult	SCZ	NCDS	0.5	-0.06	-0.13-0.01	1.09E-01	107	9.55E-03	no
Number of Rooms child vs adult	SCZ	NCDS	0.01	-0.01	-0.03-0.00	1.10E-01	108	9.64E-03	no
Father's interest in child's education	SCZ	NCDS	0.01	0.06	-0.01-0.13	1.13E-01	109	9.73E-03	no
Tenure child vs adult	MDD	NCDS	0.5	-0.06	-0.13-0.01	1.14E-01	110	9.82E-03	no
Number of Rooms Adult	SCZ	NCDS	0.3	-0.01	-0.02-0.00	1.20E-01	111	9.91E-03	no
Employment Adult	SCZ	NCDS	0.3	0.08	-0.02-0.18	1.22E-01	112	1.00E-02	no
Employment Adult	SCZ	NCDS	0.5	0.08	-0.02-0.18	1.23E-01	113	1.01E-02	no
Tenure child vs adult	SCZ	NCDS	0.3	-0.05	-0.12-0.02	1.25E-01	114	1.02E-02	no
Number of Rooms child vs adult	MDD	NCDS	0.4	0.01	-0.00-0.03	1.28E-01	115	1.03E-02	no
Smoking Adult	MDD	NCDS	1	0.06	-0.02-0.14	1.29E-01	116	1.04E-02	no
Number of Rooms child vs adult	MDD	NCDS	0.5	0.01	-0.00-0.03	1.29E-01	117	1.04E-02	no
Tenure child vs adult	SCZ	NCDS	0.4	-0.05	-0.12-0.02	1.30E-01	118	1.05E-02	no
Smoking mother prior & during pregnancy vs adult	MDD	NCDS	0.2	0.06	-0.02-0.15	1.37E-01	119	1.06E-02	no
Employment Adult	SCZ	NCDS	0.4	0.07	-0.02-0.17	1.39E-01	120	1.07E-02	no
Number of Rooms Adult	SCZ	NCDS	1	-0.01	-0.02-0.00	1.39E-01	121	1.08E-02	no
Tenure Child	MDD	NCDS	0.2	0.06	-0.02-0.14	1.41E-01	122	1.09E-02	no
Finance Issues Child	SCZ	NCDS	0.01	-0.06	-0.14-0.02	1.42E-01	123	1.10E-02	no
Tenure child vs adult	SCZ	NCDS	1	-0.05	-0.12-0.02	1.43E-01	124	1.11E-02	no
Tenure Adult	SCZ	USoc	0.3	0.05	-0.02-0.12	1.43E-01	125	1.12E-02	no
Tenure child vs adult	MDD	NCDS	0.4	-0.05	-0.12-0.02	1.43E-01	126	1.13E-02	no
Smoking Adult	MDD	NCDS	0.2	0.06	-0.02-0.13	1.45E-01	127	1.13E-02	no
Number of Rooms child vs adult	MDD	NCDS	0.2	0.01	-0.00-0.03	1.46E-01	128	1.14E-02	no
Smoking Adult	MDD	NCDS	0.3	0.06	-0.02-0.13	1.48E-01	129	1.15E-02	no
Tenure child vs adult	MDD	NCDS	1	-0.05	-0.12-0.02	1.48E-01	130	1.16E-02	no
Marital status parents vs adult	SCZ	NCDS	1	0.13	-0.05-0.31	1.49E-01	131	1.17E-02	no
Number of Rooms Child	SCZ	MCS	0.1	-0.01	-0.01-0.00	1.53E-01	132	1.18E-02	no
Tenure Adult	SCZ	USoc	1	0.05	-0.02-0.12	1.55E-01	133	1.19E-02	no
Smoking Adult	MDD	NCDS	0.5	0.06	-0.02-0.13	1.57E-01	134	1.20E-02	no
Employment Adult	MDD	NCDS	0.01	0.07	-0.03-0.17	1.57E-01	135	1.21E-02	no
Number of Rooms Adult	SCZ	NCDS	0.5	-0.01	-0.02-0.00	1.57E-01	136	1.21E-02	no
Number of Rooms Adult	SCZ	NCDS	0.2	-0.01	-0.02-0.00	1.62E-01	137	1.22E-02	no
Marital status Adult	MDD	NCDS	0.01	0.02	-0.01-0.06	1.62E-01	138	1.23E-02	no
Number of Rooms child vs adult	MDD	NCDS	1	0.01	-0.01-0.03	1.65E-01	139	1.24E-02	no
SES Child	MDD	MCS	0.01	0	-0.00-0.01	1.65E-01	140	1.25E-02	no

Tenure Adult	SCZ	USoc	0.2	0.05	-0.02-0.12	1.66E-01	141	1.26E-02	no
Smoking Adult	MDD	NCDS	0.4	0.05	-0.02-0.13	1.68E-01	142	1.27E-02	no
Finance Issues Child	SCZ	NCDS	0.1	-0.05	-0.13-0.02	1.71E-01	143	1.28E-02	no
Mother's interest in child's education	MDD	NCDS	0.3	0.04	-0.02-0.11	1.73E-01	144	1.29E-02	no
Marital status parents vs adult	SCZ	NCDS	0.5	0.12	-0.06-0.30	1.75E-01	145	1.29E-02	no
SES Child	MDD	NCDS	0.1	0.01	-0.00-0.02	1.76E-01	146	1.30E-02	no
Employment Adult	SCZ	NCDS	1	0.07	-0.03-0.17	1.80E-01	147	1.31E-02	no
Income Adult	MDD	USoc	0.3	0	-0.01-0.00	1.80E-01	148	1.32E-02	no
Number of Rooms Adult	SCZ	NCDS	0.4	-0.01	-0.02-0.00	1.84E-01	149	1.33E-02	no
Marital status parents vs adult	SCZ	NCDS	0.4	0.12	-0.06-0.30	1.84E-01	150	1.34E-02	no
Number of Rooms Child	MDD	NCDS	1	0.01	-0.00-0.02	1.87E-01	151	1.35E-02	no
Number of Rooms Child	MDD	NCDS	0.5	0.01	-0.00-0.02	1.89E-01	152	1.36E-02	no
Finance Issues Child	SCZ	NCDS	0.2	-0.05	-0.13-0.03	1.90E-01	153	1.37E-02	no
Employment Adult	SCZ	NCDS	0.2	0.07	-0.03-0.16	1.91E-01	154	1.38E-02	no
Number of Rooms Child	SCZ	MCS	0.2	-0.01	-0.01-0.00	1.93E-01	155	1.38E-02	no
Employment Adult	SCZ	NCDS	0.01	0.07	-0.04-0.17	2.01E-01	156	1.39E-02	no
Number of Rooms Adult	MDD	NCDS	0.1	-0.01	-0.02-0.00	2.02E-01	157	1.40E-02	no
Employment Adult	SCZ	NCDS	0.1	0.06	-0.03-0.16	2.03E-01	158	1.41E-02	no
Finance Issues Adult	MDD	USoc	0.4	-0.03	-0.07-0.02	2.03E-01	159	1.42E-02	no
Income Adult	MDD	USoc	0.2	0	-0.01-0.00	2.05E-01	160	1.43E-02	no
Tenure Adult	SCZ	USoc	0.4	0.05	-0.03-0.12	2.09E-01	161	1.44E-02	no
Finance Issues Adult	MDD	USoc	1	-0.03	-0.07-0.02	2.10E-01	162	1.45E-02	no
Tenure Child	MDD	MCS	0.01	-0.03	-0.08-0.02	2.12E-01	163	1.46E-02	no
Mother's interest in child's education	MDD	NCDS	1	0.04	-0.02-0.10	2.13E-01	164	1.46E-02	no
Father Reads to Child	SCZ	MCS	1	-0.08	-0.19-0.04	2.13E-01	165	1.47E-02	no
Mother's interest in child's education	MDD	NCDS	0.5	0.04	-0.02-0.10	2.14E-01	166	1.48E-02	no
Father Reads to Child	SCZ	MCS	0.5	-0.07	-0.19-0.04	2.15E-01	167	1.49E-02	no
Finance Issues Adult	MDD	USoc	0.5	-0.03	-0.07-0.02	2.15E-01	168	1.50E-02	no
Mother's interest in child's education	MDD	NCDS	0.4	0.04	-0.02-0.10	2.16E-01	169	1.51E-02	no
Number of Rooms child vs adult	MDD	NCDS	0.1	0.01	-0.01-0.03	2.16E-01	170	1.52E-02	no
Alcohol Father	MDD	MCS	1	-0.06	-0.16-0.04	2.17E-01	171	1.53E-02	no
Alcohol Father	MDD	MCS	0.3	-0.06	-0.16-0.04	2.19E-01	172	1.54E-02	no
Smoking Adult	MDD	NCDS	0.1	0.05	-0.03-0.12	2.25E-01	173	1.54E-02	no
Father Reads to Child	SCZ	MCS	0.3	-0.07	-0.19-0.04	2.26E-01	174	1.55E-02	no
Smoking mother prior & during pregnancy vs adult	SCZ	NCDS	0.01	-0.05	-0.13-0.03	2.27E-01	175	1.56E-02	no
Finance Issues Child	SCZ	NCDS	0.3	-0.05	-0.13-0.03	2.28E-01	176	1.57E-02	no

Father's interest in child's education	SCZ	NCDS	0.3	0.04	-0.03-0.11	2.29E-01	177	1.58E-02	no
Tenure Adult	SCZ	USoc	0.5	0.04	-0.03-0.11	2.31E-01	178	1.59E-02	no
Finance Issues Child	SCZ	NCDS	0.4	-0.05	-0.13-0.03	2.32E-01	179	1.60E-02	no
Smoking mother prior & during pregnancy vs adult	MDD	NCDS	0.01	0.05	-0.03-0.13	2.35E-01	180	1.61E-02	no
Finance Issues Child	MDD	MCS	0.01	-0.03	-0.09-0.02	2.40E-01	181	1.62E-02	no
Number of Rooms Child	SCZ	NCDS	0.01	0.01	-0.00-0.02	2.42E-01	182	1.63E-02	no
Father Reads to Child	SCZ	MCS	0.4	-0.07	-0.19-0.05	2.42E-01	183	1.63E-02	no
Father's interest in child's education	SCZ	NCDS	0.2	0.04	-0.03-0.11	2.42E-01	184	1.64E-02	no
Marital status Parents	SCZ	MCS	0.01	0.02	-0.01-0.04	2.44E-01	185	1.65E-02	no
Marital status parents vs adult	SCZ	NCDS	0.3	0.11	-0.07-0.28	2.49E-01	186	1.66E-02	no
Marital status Adult	SCZ	NCDS	0.2	0.02	-0.01-0.06	2.50E-01	187	1.67E-02	no
SES Child	MDD	NCDS	0.3	0.01	-0.00-0.02	2.51E-01	188	1.68E-02	no
Marital status parents vs adult	SCZ	NCDS	0.1	0.1	-0.07-0.28	2.52E-01	189	1.69E-02	no
Number of Rooms child vs adult	SCZ	NCDS	0.3	-0.01	-0.03-0.01	2.52E-01	190	1.70E-02	no
Finance Issues Child	SCZ	NCDS	1	-0.05	-0.12-0.03	2.54E-01	191	1.71E-02	no
Number of Rooms Child	MDD	NCDS	0.2	0.01	-0.00-0.02	2.54E-01	192	1.71E-02	no
Finance Issues Child	SCZ	NCDS	0.5	-0.05	-0.12-0.03	2.55E-01	193	1.72E-02	no
SES Child	MDD	NCDS	1	0.01	-0.00-0.01	2.56E-01	194	1.73E-02	no
Finance Issues Child	MDD	MCS	0.5	-0.03	-0.08-0.02	2.60E-01	195	1.74E-02	no
Father's interest in child's education	SCZ	NCDS	0.4	0.04	-0.03-0.11	2.61E-01	196	1.75E-02	no
Number of Rooms Adult	SCZ	NCDS	0.01	-0.01	-0.02-0.00	2.62E-01	197	1.76E-02	no
Number of Rooms Child	MDD	NCDS	0.4	0.01	-0.00-0.02	2.62E-01	198	1.77E-02	no
Mother's interest in child's education	MDD	NCDS	0.2	0.04	-0.03-0.10	2.63E-01	199	1.78E-02	no
Alcohol Father	MDD	MCS	0.5	-0.06	-0.15-0.04	2.64E-01	200	1.79E-02	no
Tenure child vs adult	SCZ	NCDS	0.1	-0.04	-0.11-0.03	2.65E-01	201	1.79E-02	no
Finance Issues Child	MDD	MCS	0.4	-0.03	-0.08-0.02	2.65E-01	202	1.80E-02	no
SES Child	MDD	NCDS	0.4	0.01	-0.00-0.01	2.69E-01	203	1.81E-02	no
Mother reads to Child	MDD	MCS	0.1	-0.05	-0.14-0.04	2.70E-01	204	1.82E-02	no
Tenure Child	MDD	NCDS	0.4	0.05	-0.04-0.13	2.70E-01	205	1.83E-02	no
Number of Rooms Child	SCZ	MCS	0.3	0	-0.01-0.00	2.71E-01	206	1.84E-02	no
Smoking mother prior & during pregnancy vs adult	MDD	NCDS	0.4	0.05	-0.04-0.13	2.73E-01	207	1.85E-02	no
Tenure Child	MDD	NCDS	1	0.05	-0.04-0.13	2.73E-01	208	1.86E-02	no
Tenure child vs adult	SCZ	NCDS	0.2	-0.04	-0.11-0.03	2.74E-01	209	1.87E-02	no
SES Child	MDD	NCDS	0.01	0.01	-0.00-0.01	2.75E-01	210	1.88E-02	no
Employment father vs adult	MDD	NCDS	0.01	0.07	-0.06-0.20	2.76E-01	211	1.88E-02	no
Tenure Adult	MDD	NCDS	0.01	0.03	-0.03-0.09	2.79E-01	212	1.89E-02	no

SES Child	MDD	NCDS	0.2	0.01	-0.00-0.01	2.80E-01	213	1.90E-02	no
Income Adult	MDD	USoc	0.4	0	-0.01-0.00	2.81E-01	214	1.91E-02	no
Alcohol Father	MDD	MCS	0.4	-0.05	-0.15-0.04	2.81E-01	215	1.92E-02	no
Finance Issues Child	MDD	MCS	1	-0.03	-0.08-0.02	2.82E-01	216	1.93E-02	no
Father Reads to Child	SCZ	MCS	0.2	-0.06	-0.18-0.05	2.83E-01	217	1.94E-02	no
Father's interest in child's education	MDD	NCDS	0.3	0.04	-0.03-0.11	2.83E-01	218	1.95E-02	no
Father Reads to Child	SCZ	MCS	0.1	-0.06	-0.18-0.05	2.83E-01	219	1.96E-02	no
Finance Issues Child	MDD	MCS	0.2	-0.03	-0.08-0.02	2.84E-01	220	1.96E-02	no
Alcohol Mother	MDD	MCS	0.01	-0.02	-0.05-0.01	2.84E-01	221	1.97E-02	no
Alcohol Father	MDD	MCS	0.2	-0.05	-0.15-0.04	2.85E-01	222	1.98E-02	no
Number of Rooms Adult	MDD	NCDS	0.2	-0.01	-0.02-0.01	2.87E-01	223	1.99E-02	no
Father's interest in child's education	SCZ	NCDS	1	0.04	-0.03-0.11	2.87E-01	224	2.00E-02	no
Smoking mother prior & during pregnancy vs adult	MDD	NCDS	0.5	0.04	-0.04-0.13	2.92E-01	225	2.01E-02	no
Mother walks Child	SCZ	MCS	0.2	-0.04	-0.10-0.03	2.93E-01	226	2.02E-02	no
Number of Rooms Adult	MDD	NCDS	0.3	-0.01	-0.02-0.01	2.93E-01	227	2.03E-02	no
Father's interest in child's education	MDD	NCDS	0.2	0.04	-0.03-0.11	2.96E-01	228	2.04E-02	no
Mother walks Child	SCZ	MCS	0.3	-0.04	-0.10-0.03	2.97E-01	229	2.04E-02	no
Marital status parents vs adult	SCZ	NCDS	0.2	0.1	-0.08-0.27	2.97E-01	230	2.05E-02	no
Number of Rooms Child	SCZ	MCS	0.4	0	-0.01-0.00	2.98E-01	231	2.06E-02	no
Finance Issues Child	MDD	MCS	0.3	-0.03	-0.08-0.02	3.02E-01	232	2.07E-02	no
Tenure Child	MDD	NCDS	0.5	0.04	-0.04-0.12	3.03E-01	233	2.08E-02	no
Number of Rooms Child	MDD	NCDS	0.3	0.01	-0.01-0.02	3.07E-01	234	2.09E-02	no
Father's interest in child's education	MDD	NCDS	0.1	0.04	-0.03-0.11	3.07E-01	235	2.10E-02	no
Tenure Child	MDD	NCDS	0.3	0.04	-0.04-0.12	3.08E-01	236	2.11E-02	no
Father's involvement in childcare	MDD	NCDS	0.5	0.04	-0.04-0.13	3.10E-01	237	2.12E-02	no
Mother reads to Child	MDD	MCS	0.01	-0.05	-0.14-0.05	3.12E-01	238	2.13E-02	no
Father's interest in child's education	SCZ	NCDS	0.5	0.04	-0.03-0.11	3.12E-01	239	2.13E-02	no
Smoking mother prior & during pregnancy vs adult	MDD	NCDS	1	0.04	-0.04-0.13	3.14E-01	240	2.14E-02	no
Number of Rooms Child	SCZ	MCS	0.5	0	-0.01-0.00	3.17E-01	241	2.15E-02	no
Father walks Child	SCZ	MCS	0.2	-0.05	-0.16-0.05	3.19E-01	242	2.16E-02	no
Father walks Child	SCZ	MCS	0.3	-0.05	-0.16-0.05	3.21E-01	243	2.17E-02	no
SES Child	MDD	NCDS	0.5	0	-0.00-0.01	3.22E-01	244	2.18E-02	no
Father's involvement in childcare	MDD	NCDS	1	0.04	-0.04-0.13	3.22E-01	245	2.19E-02	no
Mother walks Child	SCZ	MCS	0.4	-0.03	-0.10-0.03	3.23E-01	246	2.20E-02	no
Mother walks Child	SCZ	MCS	0.01	-0.03	-0.10-0.03	3.23E-01	247	2.21E-02	no
Mother reads to Child	MDD	MCS	1	-0.05	-0.13-0.04	3.23E-01	248	2.21E-02	no

Number of Rooms child vs adult	SCZ	NCDS	0.4	-0.01	-0.03-0.01	3.24E-01	249	2.22E-02	no
Father walks Child	SCZ	MCS	0.1	-0.05	-0.15-0.05	3.27E-01	250	2.23E-02	no
Mother walks Child	SCZ	MCS	0.1	-0.03	-0.10-0.03	3.28E-01	251	2.24E-02	no
Mother walks Child	SCZ	MCS	1	-0.03	-0.10-0.03	3.29E-01	252	2.25E-02	no
Income Adult	MDD	USoc	0.01	0	-0.01-0.00	3.30E-01	253	2.26E-02	no
Father's interest in child's education	MDD	NCDS	0.4	0.03	-0.04-0.11	3.31E-01	254	2.27E-02	no
Father walks Child	SCZ	MCS	0.4	-0.05	-0.16-0.05	3.31E-01	255	2.28E-02	no
Marital status Adult	SCZ	NCDS	0.3	0.02	-0.02-0.05	3.32E-01	256	2.29E-02	no
Mother walks Child	SCZ	MCS	0.5	-0.03	-0.10-0.03	3.33E-01	257	2.29E-02	no
Income Adult	MDD	USoc	0.5	0	-0.01-0.00	3.34E-01	258	2.30E-02	no
Employment Adult	MDD	NCDS	0.4	0.05	-0.05-0.14	3.37E-01	259	2.31E-02	no
Number of Rooms child vs adult	SCZ	NCDS	0.5	-0.01	-0.03-0.01	3.42E-01	260	2.32E-02	no
Mother's interest in child's education	MDD	NCDS	0.1	0.03	-0.03-0.09	3.45E-01	261	2.33E-02	no
Marital status Adult	SCZ	NCDS	0.4	0.02	-0.02-0.05	3.45E-01	262	2.34E-02	no
Employment Adult	MDD	USoc	0.2	0.02	-0.03-0.07	3.45E-01	263	2.35E-02	no
Number of Rooms Child	SCZ	MCS	1	0	-0.01-0.00	3.50E-01	264	2.36E-02	no
Mother reads to Child	MDD	MCS	0.5	-0.04	-0.13-0.05	3.50E-01	265	2.37E-02	no
Tenure Child	SCZ	NCDS	0.3	0.04	-0.04-0.12	3.51E-01	266	2.38E-02	no
Father's interest in child's education	MDD	NCDS	1	0.03	-0.04-0.10	3.53E-01	267	2.38E-02	no
Father walks Child	SCZ	MCS	1	-0.05	-0.16-0.06	3.58E-01	268	2.39E-02	no
Father walks	SCZ	NCDS	0.01	-0.05	-0.15-0.05	3.59E-01	269	2.40E-02	no
Marital status Adult	SCZ	NCDS	0.1	0.02	-0.02-0.05	3.60E-01	270	2.41E-02	no
Number of Rooms Adult	SCZ	NCDS	0.1	-0.01	-0.02-0.01	3.61E-01	271	2.42E-02	no
SES Adult	SCZ	USoc	0.3	0	-0.00-0.01	3.62E-01	272	2.43E-02	no
SES Adult	MDD	NCDS	0.5	0	-0.01-0.01	3.63E-01	273	2.44E-02	no
Father walks Child	SCZ	MCS	0.01	-0.04	-0.14-0.05	3.66E-01	274	2.45E-02	no
Mother walks Child	MDD	MCS	0.01	0.03	-0.04-0.10	3.69E-01	275	2.46E-02	no
Father's involvement in childcare	MDD	NCDS	0.4	0.04	-0.05-0.12	3.69E-01	276	2.46E-02	no
Income Adult	SCZ	USoc	0.2	0	-0.00-0.01	3.70E-01	277	2.47E-02	no
Employment Adult	MDD	NCDS	0.3	0.04	-0.05-0.14	3.70E-01	278	2.48E-02	no
Father walks Child	SCZ	MCS	0.5	-0.05	-0.15-0.06	3.73E-01	279	2.49E-02	no
Marital status Adult	SCZ	NCDS	1	0.02	-0.02-0.05	3.73E-01	280	2.50E-02	no
Finance Issues Child	MDD	MCS	0.1	-0.02	-0.07-0.03	3.74E-01	281	2.51E-02	no
Father's involvement in childcare	MDD	NCDS	0.2	0.04	-0.05-0.12	3.75E-01	282	2.52E-02	no
Tenure Child	SCZ	NCDS	1	0.04	-0.05-0.12	3.75E-01	283	2.53E-02	no
Marital status Adult	SCZ	NCDS	0.5	0.02	-0.02-0.05	3.75E-01	284	2.54E-02	no

Father Reads to Child	MDD	MCS	0.5	0.06	-0.07-0.18	3.76E-01	285	2.54E-02	no
SES Adult	SCZ	USoc	0.4	0	-0.00-0.01	3.77E-01	286	2.55E-02	no
Employment Adult	MDD	NCDS	0.5	0.04	-0.05-0.14	3.78E-01	287	2.56E-02	no
Finance Issues Adult	MDD	USoc	0.3	-0.02	-0.06-0.02	3.78E-01	288	2.57E-02	no
Employment Adult	MDD	NCDS	0.1	0.04	-0.05-0.14	3.78E-01	289	2.58E-02	no
Father's interest in child's education	MDD	NCDS	0.5	0.03	-0.04-0.10	3.79E-01	290	2.59E-02	no
Father's involvement in childcare	MDD	NCDS	0.3	0.04	-0.05-0.12	3.84E-01	291	2.60E-02	no
Employment Adult	MDD	NCDS	0.2	0.04	-0.05-0.14	3.88E-01	292	2.61E-02	no
Income Adult	MDD	USoc	1	0	-0.01-0.00	3.88E-01	293	2.62E-02	no
Smoking mother prior & during pregnancy vs adult	MDD	NCDS	0.1	0.04	-0.05-0.12	3.89E-01	294	2.63E-02	no
SES Adult	SCZ	USoc	0.2	0	-0.00-0.01	3.90E-01	295	2.63E-02	no
Marital status Parents	SCZ	MCS	0.1	0.01	-0.01-0.04	3.91E-01	296	2.64E-02	no
Tenure Child	SCZ	NCDS	0.5	0.04	-0.05-0.12	3.93E-01	297	2.65E-02	no
Number of Rooms child vs adult	SCZ	NCDS	1	-0.01	-0.03-0.01	3.95E-01	298	2.66E-02	no
Father Reads to Child	MDD	MCS	0.4	0.05	-0.07-0.18	3.96E-01	299	2.67E-02	no
Employment Adult	MDD	USoc	0.3	0.02	-0.03-0.07	3.96E-01	300	2.68E-02	no
Employment Adult	SCZ	USoc	0.5	-0.02	-0.07-0.03	4.02E-01	301	2.69E-02	no
SES Adult	MDD	NCDS	0.4	0	-0.01-0.01	4.07E-01	302	2.70E-02	no
Mother walks	MDD	NCDS	0.01	0.07	-0.10-0.23	4.09E-01	303	2.71E-02	no
SES Adult	SCZ	USoc	1	0	-0.00-0.01	4.10E-01	304	2.71E-02	no
SES Adult	SCZ	USoc	0.5	0	-0.00-0.01	4.15E-01	305	2.72E-02	no
Father walks	SCZ	NCDS	0.4	0.04	-0.06-0.14	4.15E-01	306	2.73E-02	no
Father walks	SCZ	NCDS	0.2	0.04	-0.06-0.14	4.16E-01	307	2.74E-02	no
Tenure Child	MDD	NCDS	0.01	0.03	-0.05-0.12	4.16E-01	308	2.75E-02	no
Tenure Child	SCZ	NCDS	0.1	0.03	-0.05-0.12	4.17E-01	309	2.76E-02	no
Alcohol Father	MDD	MCS	0.1	-0.04	-0.13-0.05	4.17E-01	310	2.77E-02	no
Marital status Parents	SCZ	MCS	0.3	0.01	-0.02-0.04	4.21E-01	311	2.78E-02	no
Employment Adult	SCZ	USoc	1	-0.02	-0.07-0.03	4.21E-01	312	2.79E-02	no
Mother reads to Child	MDD	MCS	0.4	-0.04	-0.13-0.05	4.22E-01	313	2.79E-02	no
Mother reads to Child	SCZ	MCS	1	-0.04	-0.13-0.06	4.23E-01	314	2.80E-02	no
SES Adult	MDD	NCDS	0.3	0	-0.01-0.01	4.24E-01	315	2.81E-02	no
Mother reads to Child	MDD	MCS	0.3	-0.04	-0.13-0.05	4.24E-01	316	2.82E-02	no
Tenure Child	SCZ	NCDS	0.4	0.03	-0.05-0.12	4.27E-01	317	2.83E-02	no
Tenure Child	MDD	NCDS	0.1	0.03	-0.05-0.11	4.28E-01	318	2.84E-02	no
Marital status Parents	SCZ	MCS	1	0.01	-0.02-0.04	4.31E-01	319	2.85E-02	no
Marital status Parents	SCZ	MCS	0.5	0.01	-0.02-0.04	4.31E-01	320	2.86E-02	no

Father Reads to Child	MDD	MCS	0.3	0.05	-0.07-0.17	4.34E-01	321	2.87E-02	no
Mother reads to Child	SCZ	MCS	0.5	-0.04	-0.13-0.06	4.37E-01	322	2.88E-02	no
Tenure Adult	SCZ	NCDS	0.01	-0.02	-0.08-0.03	4.39E-01	323	2.88E-02	no
Marital status Parents	SCZ	MCS	0.4	0.01	-0.02-0.04	4.43E-01	324	2.89E-02	no
Employment father vs adult	SCZ	NCDS	0.01	0.05	-0.08-0.17	4.43E-01	325	2.90E-02	no
Finance Issues Adult	MDD	USoc	0.01	-0.02	-0.06-0.03	4.43E-01	326	2.91E-02	no
SES Adult	MDD	NCDS	1	0	-0.01-0.01	4.44E-01	327	2.92E-02	no
Marital status Parents	MDD	MCS	0.2	-0.01	-0.04-0.02	4.45E-01	328	2.93E-02	no
Father walks	MDD	NCDS	0.3	-0.04	-0.14-0.06	4.45E-01	329	2.94E-02	no
Father walks	SCZ	NCDS	0.3	0.04	-0.06-0.14	4.49E-01	330	2.95E-02	no
Mother reads to Child	MDD	MCS	0.2	-0.04	-0.13-0.06	4.49E-01	331	2.96E-02	no
Marital status Parents	SCZ	MCS	0.2	0.01	-0.02-0.04	4.50E-01	332	2.96E-02	no
Father walks	SCZ	NCDS	0.5	0.04	-0.06-0.14	4.51E-01	333	2.97E-02	no
Number of Rooms child vs adult	SCZ	NCDS	0.2	-0.01	-0.03-0.01	4.51E-01	334	2.98E-02	no
Father walks	MDD	NCDS	0.5	-0.04	-0.14-0.06	4.51E-01	335	2.99E-02	no
Mother's interest in child's education	MDD	NCDS	0.01	0.02	-0.04-0.09	4.52E-01	336	3.00E-02	no
Number of Rooms Adult	MDD	NCDS	0.4	0	-0.02-0.01	4.53E-01	337	3.01E-02	no
Tenure Child	SCZ	NCDS	0.2	0.03	-0.05-0.11	4.54E-01	338	3.02E-02	no
Mother reads to Child	SCZ	MCS	0.4	-0.04	-0.13-0.06	4.56E-01	339	3.03E-02	no
Employment Adult	SCZ	USoc	0.01	-0.02	-0.07-0.03	4.59E-01	340	3.04E-02	no
Employment Adult	MDD	NCDS	1	0.04	-0.06-0.13	4.67E-01	341	3.04E-02	no
Finance Issues Adult	SCZ	USoc	1	-0.02	-0.06-0.03	4.68E-01	342	3.05E-02	no
Smoking mother prior & during pregnancy vs adult	SCZ	NCDS	0.1	-0.03	-0.11-0.05	4.68E-01	343	3.06E-02	no
Marital status Parents	MDD	MCS	0.4	-0.01	-0.04-0.02	4.69E-01	344	3.07E-02	no
Father Reads to Child	MDD	MCS	0.01	-0.04	-0.14-0.06	4.70E-01	345	3.08E-02	no
Income Adult	SCZ	USoc	0.1	0	-0.00-0.01	4.72E-01	346	3.09E-02	no
Father walks	SCZ	NCDS	1	0.04	-0.06-0.14	4.73E-01	347	3.10E-02	no
Mother reads to Child	SCZ	MCS	0.2	-0.03	-0.13-0.06	4.73E-01	348	3.11E-02	no
Employment Adult	MDD	USoc	1	0.02	-0.03-0.07	4.75E-01	349	3.12E-02	no
Father walks	SCZ	NCDS	0.1	0.04	-0.06-0.14	4.79E-01	350	3.13E-02	no
Marital status Parents	MDD	MCS	0.3	-0.01	-0.04-0.02	4.81E-01	351	3.13E-02	no
Alcohol Father	SCZ	MCS	0.01	0.03	-0.05-0.10	4.82E-01	352	3.14E-02	no
SES Child	SCZ	NCDS	0.1	0	-0.01-0.01	4.82E-01	353	3.15E-02	no
Father's involvement in childcare	MDD	NCDS	0.1	0.03	-0.05-0.11	4.86E-01	354	3.16E-02	no
Finance Issues Adult	SCZ	USoc	0.4	-0.02	-0.06-0.03	4.87E-01	355	3.17E-02	no
Finance Issues Adult	SCZ	USoc	0.1	-0.02	-0.06-0.03	4.92E-01	356	3.18E-02	no

Smoking Mother	SCZ	MCS	0.01	0.02	-0.03-0.06	4.94E-01	357	3.19E-02	no
Father walks	MDD	NCDS	0.4	-0.03	-0.13-0.07	4.95E-01	358	3.20E-02	no
Mother reads to Child	SCZ	MCS	0.3	-0.03	-0.13-0.06	4.96E-01	359	3.21E-02	no
Employment Adult	SCZ	USoc	0.1	-0.02	-0.07-0.03	5.01E-01	360	3.21E-02	no
Father Reads to Child	MDD	MCS	1	0.04	-0.08-0.16	5.01E-01	361	3.22E-02	no
Father walks	MDD	NCDS	1	-0.03	-0.13-0.07	5.11E-01	362	3.23E-02	no
Tenure Child	MDD	MCS	1	0.02	-0.03-0.06	5.14E-01	363	3.24E-02	no
Marital status parents vs adult	SCZ	NCDS	0.01	0.06	-0.12-0.24	5.19E-01	364	3.25E-02	no
SES Child	SCZ	NCDS	0.3	0	-0.01-0.01	5.19E-01	365	3.26E-02	no
Employment Adult	MDD	USoc	0.4	0.02	-0.03-0.06	5.22E-01	366	3.27E-02	no
Income Adult	SCZ	USoc	0.3	0	-0.00-0.01	5.24E-01	367	3.28E-02	no
Number of Rooms Child	MDD	NCDS	0.1	0	-0.01-0.01	5.24E-01	368	3.29E-02	no
Smoking Adult	SCZ	NCDS	0.1	0.02	-0.05-0.10	5.25E-01	369	3.29E-02	no
Marital status parents vs adult	MDD	NCDS	0.01	-0.06	-0.24-0.12	5.25E-01	370	3.30E-02	no
Marital status Parents	MDD	MCS	0.01	-0.01	-0.03-0.02	5.27E-01	371	3.31E-02	no
Employment Father Child	MDD	NCDS	0.1	0.04	-0.08-0.16	5.28E-01	372	3.32E-02	no
Alcohol Father	MDD	MCS	0.01	-0.02	-0.09-0.05	5.29E-01	373	3.33E-02	no
Employment Adult	SCZ	USoc	0.4	-0.02	-0.07-0.03	5.30E-01	374	3.34E-02	no
Employment Adult	MDD	USoc	0.5	0.02	-0.03-0.06	5.31E-01	375	3.35E-02	no
Number of Rooms Adult	MDD	NCDS	0.5	0	-0.01-0.01	5.32E-01	376	3.36E-02	no
SES Child	SCZ	NCDS	1	0	-0.01-0.01	5.32E-01	377	3.37E-02	no
SES Adult	MDD	NCDS	0.2	0	-0.01-0.01	5.33E-01	378	3.38E-02	no
Smoking Adult	SCZ	NCDS	0.3	0.02	-0.05-0.10	5.40E-01	379	3.38E-02	no
Mother reads to Child	SCZ	MCS	0.1	-0.03	-0.13-0.07	5.41E-01	380	3.39E-02	no
Father Reads to Child	MDD	MCS	0.2	0.04	-0.08-0.16	5.42E-01	381	3.40E-02	no
Alcohol Mother	MDD	MCS	0.2	-0.01	-0.06-0.03	5.43E-01	382	3.41E-02	no
Marital status Parents	MDD	MCS	0.5	-0.01	-0.04-0.02	5.46E-01	383	3.42E-02	no
Finance Issues Adult	SCZ	USoc	0.2	-0.01	-0.06-0.03	5.46E-01	384	3.43E-02	no
Father's involvement in childcare	SCZ	NCDS	0.2	-0.03	-0.11-0.06	5.50E-01	385	3.44E-02	no
Alcohol Father	SCZ	MCS	0.4	-0.03	-0.11-0.06	5.53E-01	386	3.45E-02	no
Number of Rooms Adult	MDD	NCDS	1	0	-0.01-0.01	5.55E-01	387	3.46E-02	no
Tenure Child	MDD	MCS	0.1	-0.01	-0.06-0.03	5.60E-01	388	3.46E-02	no
Marital status parents vs adult	MDD	NCDS	0.5	-0.05	-0.24-0.13	5.62E-01	389	3.47E-02	no
Finance Issues Adult	SCZ	USoc	0.5	-0.01	-0.06-0.03	5.63E-01	390	3.48E-02	no
Tenure Adult	MDD	NCDS	1	-0.02	-0.07-0.04	5.67E-01	391	3.49E-02	no
Income Adult	SCZ	USoc	1	0	-0.00-0.01	5.71E-01	392	3.50E-02	no

Finance Issues Adult	MDD	USoc	0.2	-0.01	-0.06-0.03	5.77E-01	393	3.51E-02	no
Smoking mother prior & during pregnancy vs adult	SCZ	NCDS	0.5	-0.02	-0.11-0.06	5.79E-01	394	3.52E-02	no
SES Child	SCZ	NCDS	0.5	0	-0.01-0.01	5.82E-01	395	3.53E-02	no
Tenure Child	MDD	MCS	0.5	0.01	-0.03-0.06	5.90E-01	396	3.54E-02	no
Alcohol Mother	MDD	MCS	0.1	-0.01	-0.05-0.03	5.94E-01	397	3.54E-02	no
SES Child	SCZ	NCDS	0.4	0	-0.01-0.01	5.98E-01	398	3.55E-02	no
Smoking mother prior & during pregnancy vs adult	SCZ	NCDS	1	-0.02	-0.11-0.06	5.98E-01	399	3.56E-02	no
Employment father vs adult	SCZ	NCDS	0.2	-0.03	-0.16-0.09	6.00E-01	400	3.57E-02	no
Finance Issues Adult	SCZ	USoc	0.3	-0.01	-0.06-0.03	6.04E-01	401	3.58E-02	no
Alcohol Father	SCZ	MCS	0.5	-0.02	-0.11-0.06	6.07E-01	402	3.59E-02	no
Marital status Parents	MDD	MCS	0.1	-0.01	-0.03-0.02	6.08E-01	403	3.60E-02	no
Alcohol Father	SCZ	MCS	1	-0.02	-0.11-0.07	6.12E-01	404	3.61E-02	no
Mother's interest in child's education	SCZ	NCDS	0.1	0.02	-0.05-0.08	6.12E-01	405	3.62E-02	no
Tenure Adult	MDD	NCDS	0.2	-0.01	-0.07-0.04	6.12E-01	406	3.63E-02	no
Father walks	MDD	NCDS	0.1	-0.03	-0.13-0.07	6.16E-01	407	3.63E-02	no
Marital status Parents	MDD	MCS	1	-0.01	-0.03-0.02	6.18E-01	408	3.64E-02	no
Alcohol Mother	MDD	MCS	0.5	-0.01	-0.05-0.03	6.19E-01	409	3.65E-02	no
Alcohol Father	SCZ	MCS	0.3	-0.02	-0.11-0.07	6.19E-01	410	3.66E-02	no
Father's involvement in childcare	SCZ	NCDS	0.3	-0.02	-0.11-0.06	6.20E-01	411	3.67E-02	no
Income Adult	MDD	USoc	0.1	0	-0.01-0.00	6.21E-01	412	3.68E-02	no
Tenure Child	MDD	MCS	0.4	0.01	-0.03-0.06	6.21E-01	413	3.69E-02	no
Alcohol Mother	MDD	MCS	0.4	-0.01	-0.05-0.03	6.21E-01	414	3.70E-02	no
Income Adult	SCZ	USoc	0.4	0	-0.00-0.01	6.22E-01	415	3.71E-02	no
SES Adult	MDD	NCDS	0.01	0	-0.01-0.01	6.27E-01	416	3.71E-02	no
Marital status parents vs adult	MDD	NCDS	0.4	-0.05	-0.23-0.14	6.28E-01	417	3.72E-02	no
Tenure Adult	MDD	NCDS	0.3	-0.01	-0.07-0.04	6.29E-01	418	3.73E-02	no
Father's interest in child's education	MDD	NCDS	0.01	0.02	-0.05-0.09	6.32E-01	419	3.74E-02	no
Smoking Mother	MDD	MCS	0.01	0.01	-0.03-0.05	6.32E-01	420	3.75E-02	no
Mother walks	MDD	NCDS	0.4	-0.04	-0.21-0.13	6.33E-01	421	3.76E-02	no
Alcohol Mother	MDD	MCS	1	-0.01	-0.05-0.03	6.33E-01	422	3.77E-02	no
Smoking Adult	SCZ	NCDS	0.4	0.02	-0.06-0.09	6.33E-01	423	3.78E-02	no
Marital status parents vs adult	MDD	NCDS	1	-0.04	-0.23-0.14	6.35E-01	424	3.79E-02	no
Smoking mother prior & during pregnancy vs adult	SCZ	NCDS	0.4	-0.02	-0.10-0.06	6.36E-01	425	3.79E-02	no
Alcohol Mother	MDD	MCS	0.3	-0.01	-0.05-0.03	6.36E-01	426	3.80E-02	no
Father Reads to Child	MDD	MCS	0.1	0.03	-0.09-0.15	6.39E-01	427	3.81E-02	no
Tenure Adult	MDD	NCDS	0.5	-0.01	-0.07-0.04	6.39E-01	428	3.82E-02	no

SES Child	SCZ	NCDS	0.01	0	-0.01-0.01	6.42E-01	429	3.83E-02	no
Smoking mother prior & during pregnancy vs adult	SCZ	NCDS	0.3	-0.02	-0.10-0.06	6.49E-01	430	3.84E-02	no
Income Adult	SCZ	USoc	0.5	0	-0.00-0.01	6.55E-01	431	3.85E-02	no
Tenure Child	MDD	MCS	0.3	0.01	-0.04-0.06	6.58E-01	432	3.86E-02	no
SES Child	SCZ	NCDS	0.2	0	-0.01-0.01	6.62E-01	433	3.87E-02	no
Mother walks	MDD	NCDS	0.5	-0.04	-0.20-0.13	6.63E-01	434	3.88E-02	no
SES Adult	SCZ	USoc	0.01	0	-0.00-0.01	6.63E-01	435	3.88E-02	no
Employment Father Child	MDD	NCDS	0.01	0.03	-0.12-0.19	6.64E-01	436	3.89E-02	no
Number of Rooms Child	MDD	MCS	1	0	-0.01-0.01	6.65E-01	437	3.90E-02	no
Tenure Child	MDD	MCS	0.2	0.01	-0.04-0.06	6.70E-01	438	3.91E-02	no
Mother's interest in child's education	SCZ	NCDS	0.4	0.01	-0.05-0.08	6.72E-01	439	3.92E-02	no
Finance Issues Adult	SCZ	USoc	0.01	0.01	-0.04-0.05	6.76E-01	440	3.93E-02	no
Father's involvement in childcare	SCZ	NCDS	0.1	-0.02	-0.10-0.07	6.78E-01	441	3.94E-02	no
Tenure Adult	MDD	NCDS	0.4	-0.01	-0.07-0.04	6.79E-01	442	3.95E-02	no
Employment Father Child	SCZ	NCDS	0.5	0.03	-0.12-0.18	6.82E-01	443	3.96E-02	no
Employment Adult	MDD	USoc	0.1	0.01	-0.04-0.06	6.84E-01	444	3.96E-02	no
Smoking Mother	SCZ	MCS	0.4	0.01	-0.04-0.06	6.86E-01	445	3.97E-02	no
Employment Adult	SCZ	USoc	0.2	-0.01	-0.06-0.04	6.87E-01	446	3.98E-02	no
Mother walks	MDD	NCDS	0.3	-0.03	-0.20-0.13	6.93E-01	447	3.99E-02	no
Smoking Mother	SCZ	MCS	0.3	0.01	-0.04-0.05	6.97E-01	448	4.00E-02	no
Employment Father Child	SCZ	NCDS	0.3	0.03	-0.12-0.18	7.05E-01	449	4.01E-02	no
Mother's interest in child's education	SCZ	NCDS	0.3	0.01	-0.05-0.08	7.06E-01	450	4.02E-02	no
Smoking Adult	SCZ	NCDS	0.5	0.01	-0.06-0.09	7.07E-01	451	4.03E-02	no
Finance Issues Child	MDD	NCDS	0.1	-0.01	-0.09-0.06	7.07E-01	452	4.04E-02	no
Employment father vs adult	SCZ	NCDS	0.3	-0.02	-0.15-0.10	7.08E-01	453	4.04E-02	no
Smoking Mother	SCZ	MCS	0.2	0.01	-0.04-0.05	7.09E-01	454	4.05E-02	no
Employment Father Child	SCZ	NCDS	0.4	0.03	-0.12-0.18	7.12E-01	455	4.06E-02	no
Father walks	MDD	NCDS	0.2	-0.02	-0.12-0.08	7.14E-01	456	4.07E-02	no
Smoking Mother	SCZ	MCS	0.5	0.01	-0.04-0.05	7.14E-01	457	4.08E-02	no
Mother reads to Child	SCZ	MCS	0.01	-0.02	-0.11-0.08	7.15E-01	458	4.09E-02	no
Smoking Adult	SCZ	NCDS	1	0.01	-0.06-0.09	7.16E-01	459	4.10E-02	no
Mother walks	MDD	NCDS	1	-0.03	-0.20-0.14	7.17E-01	460	4.11E-02	no
Smoking Adult	SCZ	NCDS	0.2	0.01	-0.06-0.09	7.19E-01	461	4.12E-02	no
Alcohol Mother	SCZ	MCS	1	-0.01	-0.04-0.03	7.23E-01	462	4.13E-02	no
Employment father vs adult	SCZ	NCDS	0.1	-0.02	-0.15-0.10	7.24E-01	463	4.13E-02	no
Father walks Child	MDD	MCS	0.01	0.02	-0.08-0.11	7.29E-01	464	4.14E-02	no

Employment Father Child	SCZ	NCDS	1	0.03	-0.13-0.18	7.30E-01	465	4.15E-02	no
Mother walks Child	MDD	MCS	0.3	0.01	-0.06-0.08	7.33E-01	466	4.16E-02	no
Smoking Mother	SCZ	MCS	1	0.01	-0.04-0.05	7.36E-01	467	4.17E-02	no
Employment Adult	SCZ	USoc	0.3	-0.01	-0.06-0.04	7.46E-01	468	4.18E-02	no
Employment Father Child	MDD	NCDS	0.5	-0.03	-0.18-0.13	7.47E-01	469	4.19E-02	no
Employment father vs adult	SCZ	NCDS	1	-0.02	-0.15-0.11	7.48E-01	470	4.20E-02	no
Employment Father Child	MDD	NCDS	0.4	-0.03	-0.18-0.13	7.53E-01	471	4.21E-02	no
Smoking Mother	MDD	MCS	0.1	0.01	-0.04-0.05	7.55E-01	472	4.21E-02	no
Smoking Mother	MDD	MCS	0.5	0.01	-0.04-0.05	7.55E-01	473	4.22E-02	no
Number of Rooms Child	MDD	MCS	0.5	0	-0.01-0.01	7.58E-01	474	4.23E-02	no
Finance Issues Child	SCZ	MCS	0.1	-0.01	-0.06-0.04	7.61E-01	475	4.24E-02	no
Smoking mother prior & during pregnancy vs adult	SCZ	NCDS	0.2	-0.01	-0.10-0.07	7.61E-01	476	4.25E-02	no
Income Adult	SCZ	USoc	0.01	0	-0.00-0.01	7.64E-01	477	4.26E-02	no
Alcohol Father	SCZ	MCS	0.2	-0.01	-0.10-0.07	7.66E-01	478	4.27E-02	no
Finance Issues Child	MDD	NCDS	0.2	0.01	-0.07-0.09	7.67E-01	479	4.28E-02	no
Employment Father Child	SCZ	NCDS	0.1	-0.02	-0.17-0.13	7.69E-01	480	4.29E-02	no
Mother walks	SCZ	NCDS	0.01	0.02	-0.14-0.19	7.71E-01	481	4.29E-02	no
Alcohol Mother	SCZ	MCS	0.5	-0.01	-0.04-0.03	7.72E-01	482	4.30E-02	no
Employment Father Child	SCZ	NCDS	0.01	0.02	-0.13-0.17	7.74E-01	483	4.31E-02	no
Alcohol Mother	SCZ	MCS	0.4	-0.01	-0.04-0.03	7.75E-01	484	4.32E-02	no
Father's involvement in childcare	MDD	NCDS	0.01	-0.01	-0.10-0.07	7.75E-01	485	4.33E-02	no
Number of Rooms Child	MDD	MCS	0.4	0	-0.01-0.01	7.78E-01	486	4.34E-02	no
Finance Issues Adult	MDD	USoc	0.1	-0.01	-0.05-0.04	7.80E-01	487	4.35E-02	no
Finance Issues Child	SCZ	MCS	0.5	-0.01	-0.06-0.04	7.81E-01	488	4.36E-02	no
Smoking Mother	MDD	MCS	0.4	0.01	-0.04-0.05	7.86E-01	489	4.37E-02	no
Employment Adult	MDD	USoc	0.01	0.01	-0.04-0.06	7.88E-01	490	4.38E-02	no
Employment father vs adult	SCZ	NCDS	0.5	-0.02	-0.14-0.11	7.90E-01	491	4.38E-02	no
Smoking Mother	MDD	MCS	1	0.01	-0.04-0.05	7.90E-01	492	4.39E-02	no
Smoking Mother	MDD	MCS	0.3	0.01	-0.04-0.05	7.94E-01	493	4.40E-02	no
Marital status parents vs adult	MDD	NCDS	0.3	-0.02	-0.21-0.16	7.94E-01	494	4.41E-02	no
Smoking Mother	MDD	MCS	0.2	0.01	-0.04-0.05	7.95E-01	495	4.42E-02	no
Finance Issues Child	SCZ	MCS	1	-0.01	-0.06-0.04	7.96E-01	496	4.43E-02	no
Number of Rooms Adult	MDD	NCDS	0.01	0	-0.01-0.01	7.99E-01	497	4.44E-02	no
Father's involvement in childcare	SCZ	NCDS	0.4	-0.01	-0.09-0.07	8.01E-01	498	4.45E-02	no
Mother walks Child	MDD	MCS	0.2	0.01	-0.06-0.08	8.02E-01	499	4.46E-02	no
Smoking Mother	SCZ	MCS	0.1	0.01	-0.04-0.05	8.04E-01	500	4.46E-02	no

Alcohol Mother	SCZ	MCS	0.3	0	-0.04-0.03	8.05E-01	501	4.47E-02	no
Finance Issues Child	SCZ	MCS	0.4	-0.01	-0.06-0.04	8.05E-01	502	4.48E-02	no
Employment Father Child	MDD	NCDS	0.2	-0.02	-0.18-0.14	8.07E-01	503	4.49E-02	no
Tenure Child	SCZ	NCDS	0.01	0.01	-0.07-0.09	8.07E-01	504	4.50E-02	no
Employment father vs adult	SCZ	NCDS	0.4	-0.02	-0.14-0.11	8.09E-01	505	4.51E-02	no
Alcohol Mother	SCZ	MCS	0.01	0	-0.04-0.03	8.12E-01	506	4.52E-02	no
Mother's interest in child's education	SCZ	NCDS	0.5	0.01	-0.06-0.07	8.17E-01	507	4.53E-02	no
Mother's interest in child's education	SCZ	NCDS	0.2	0.01	-0.06-0.07	8.19E-01	508	4.54E-02	no
SES Adult	SCZ	USoc	0.1	0	-0.00-0.01	8.22E-01	509	4.54E-02	no
Mother walks Child	MDD	MCS	0.1	0.01	-0.06-0.08	8.23E-01	510	4.55E-02	no
Alcohol Mother	SCZ	MCS	0.2	0	-0.04-0.03	8.23E-01	511	4.56E-02	no
Number of Rooms Child	MDD	MCS	0.3	0	-0.01-0.01	8.25E-01	512	4.57E-02	no
SES Adult	MDD	NCDS	0.1	0	-0.01-0.01	8.27E-01	513	4.58E-02	no
Father walks Child	MDD	MCS	0.1	-0.01	-0.12-0.10	8.27E-01	514	4.59E-02	no
Finance Issues Child	MDD	NCDS	0.3	0.01	-0.07-0.09	8.36E-01	515	4.60E-02	no
Alcohol Mother	SCZ	MCS	0.1	0	-0.04-0.03	8.39E-01	516	4.61E-02	no
Mother walks	MDD	NCDS	0.1	0.02	-0.15-0.18	8.43E-01	517	4.62E-02	no
Finance Issues Child	SCZ	MCS	0.3	-0.01	-0.05-0.04	8.43E-01	518	4.63E-02	no
Finance Issues Child	SCZ	MCS	0.01	-0.01	-0.06-0.05	8.49E-01	519	4.63E-02	no
Finance Issues Child	SCZ	MCS	0.2	0	-0.05-0.05	8.50E-01	520	4.64E-02	no
Employment Father Child	MDD	NCDS	1	-0.01	-0.17-0.14	8.55E-01	521	4.65E-02	no
Mother walks Child	MDD	MCS	0.4	0.01	-0.06-0.07	8.69E-01	522	4.66E-02	no
Father's involvement in childcare	SCZ	NCDS	0.5	-0.01	-0.09-0.08	8.73E-01	523	4.67E-02	no
Father's involvement in childcare	SCZ	NCDS	1	-0.01	-0.09-0.08	8.75E-01	524	4.68E-02	no
Father's involvement in childcare	SCZ	NCDS	0.01	-0.01	-0.09-0.08	8.75E-01	525	4.69E-02	no
Mother walks	SCZ	NCDS	0.1	0.01	-0.15-0.18	8.76E-01	526	4.70E-02	no
Mother walks	SCZ	NCDS	0.4	0.01	-0.15-0.18	8.77E-01	527	4.71E-02	no
Mother walks	SCZ	NCDS	0.2	0.01	-0.15-0.18	8.80E-01	528	4.71E-02	no
Smoking Adult	SCZ	NCDS	0.01	-0.01	-0.08-0.07	8.90E-01	529	4.72E-02	no
Mother walks	SCZ	NCDS	0.5	0.01	-0.15-0.18	8.96E-01	530	4.73E-02	no
Mother's interest in child's education	SCZ	NCDS	1	0	-0.06-0.07	9.01E-01	531	4.74E-02	no
Father walks Child	MDD	MCS	0.5	0.01	-0.11-0.12	9.04E-01	532	4.75E-02	no
Number of Rooms child vs adult	SCZ	NCDS	0.1	0	-0.02-0.02	9.06E-01	533	4.76E-02	no
Father walks Child	MDD	MCS	0.4	0.01	-0.11-0.12	9.06E-01	534	4.77E-02	no
Mother's interest in child's education	SCZ	NCDS	0.01	0	-0.06-0.07	9.09E-01	535	4.78E-02	no
Number of Rooms Child	MDD	MCS	0.2	0	-0.01-0.01	9.11E-01	536	4.79E-02	no

Mother walks	SCZ	NCDS	1	-0.01	-0.17-0.16	9.18E-01	537	4.79E-02	no
Father walks	MDD	NCDS	0.01	0	-0.10-0.10	9.31E-01	538	4.80E-02	no
Father walks Child	MDD	MCS	0.3	0	-0.11-0.12	9.35E-01	539	4.81E-02	no
Father walks Child	MDD	MCS	0.2	0	-0.11-0.12	9.37E-01	540	4.82E-02	no
Tenure Adult	MDD	NCDS	0.1	0	-0.05-0.06	9.38E-01	541	4.83E-02	no
Finance Issues Child	MDD	NCDS	1	0	-0.08-0.08	9.43E-01	542	4.84E-02	no
Number of Rooms Child	MDD	NCDS	0.01	0	-0.01-0.01	9.44E-01	543	4.85E-02	no
Mother walks Child	MDD	MCS	1	0	-0.07-0.07	9.45E-01	544	4.86E-02	no
Marital status parents vs adult	MDD	NCDS	0.1	0.01	-0.18-0.19	9.45E-01	545	4.87E-02	no
Employment Father Child	SCZ	NCDS	0.2	-0.01	-0.16-0.15	9.47E-01	546	4.88E-02	no
SES child vs adult	SCZ	NCDS	0.01	0	-0.02-0.02	9.54E-01	547	4.88E-02	no
Mother walks	SCZ	NCDS	0.3	0	-0.16-0.17	9.58E-01	548	4.89E-02	no
Number of Rooms Child	MDD	MCS	0.01	0	-0.01-0.01	9.60E-01	549	4.90E-02	no
Father walks Child	MDD	MCS	1	0	-0.11-0.11	9.65E-01	550	4.91E-02	no
Finance Issues Child	MDD	NCDS	0.01	0	-0.08-0.08	9.67E-01	551	4.92E-02	no
Mother walks	MDD	NCDS	0.2	0	-0.16-0.17	9.71E-01	552	4.93E-02	no
Alcohol Father	SCZ	MCS	0.1	0	-0.08-0.08	9.73E-01	553	4.94E-02	no
Tenure child vs adult	SCZ	NCDS	0.01	0	-0.07-0.07	9.77E-01	554	4.95E-02	no
Finance Issues Child	MDD	NCDS	0.5	0	-0.08-0.08	9.78E-01	555	4.96E-02	no
Number of Rooms Child	MDD	MCS	0.1	0	-0.01-0.01	9.81E-01	556	4.96E-02	no
Employment Father Child	MDD	NCDS	0.3	0	-0.16-0.16	9.81E-01	557	4.97E-02	no
Finance Issues Child	MDD	NCDS	0.4	0	-0.08-0.08	9.85E-01	558	4.98E-02	no
Mother walks Child	MDD	MCS	0.5	0	-0.07-0.07	9.86E-01	559	4.99E-02	no
Marital status parents vs adult	MDD	NCDS	0.2	0	-0.18-0.18	9.99E-01	560	5.00E-02	no

Note: Benjamini-Hochberg correction adjusted α=(rank of p-value/number of tests for each threshold) - *α [adjusted α=(rank/560)*0.05]. Beta = beta coefficient, CI = Confidence Interval, adj alpha = adjusted alpha, sig? = significant

References

1. StataCorp. *Stata Statistical Software: Release 12*. College Station, TX: StataCorp LP; 2011.
