# Supplementary material for: Gene–Environment Correlation over Time: A Longitudinal Analysis of Polygenic Risk Scores for Schizophrenia and Major Depression in Three British Cohorts Studies
Source: Genes (Basel). 2022 Jun 24;13(7):1136. doi: 10.3390/genes13071136 (PMC9320197; doi:10.3390/genes13071136)
Supplement: Supplementary file 1 [file genes-13-01136-s001.zip › Supplementary_S5_Cohort_characteristics.pdf]

Supplementary Document S5– Cohort Characteristics

Table of Contents

Table S14: Cohort Characteristics for MCS 2
Table S15: Cohort Characteristics for USoc 6
Table S16: Cohort Characteristics for NCDS - childhood 10
Table S17: Cohort Characteristics for NCDS – adulthood 14
References 16

All descriptive statistics were calculated using STATA v12.1 (StataCorp, 2011).

Table S14: Cohort Characteristics for MCS

Environment	Wave	MCS Total			
		Number of participants	% Participants	Mean	(SD)
**Age**	N/A	aged 9 months (wave 1 in 2001) to 14 years (wave 6 in 2015)			
**Participants**	N/A	18,476	100	N/A	N/A
children		7,280	39.40		
mothers		6,874	37.21		
fathers		4,322	23.39		
**Gender - children**	N/A	6,943	100	N/A	N/A
Males		3,401	48.98		
Females		3,542	51.02		
**Mother takes child for walks/park**	3	6,384	100.00	N/A	N/A
Yes		5,650	88.50		
No		734	11.50		
**Mother takes child for walks/park**	4	6,257	100.00	N/A	N/A
Yes		5,120	81.83		
No		1,137	18.17		
**Father takes child for walks/park**	3	3,852	100.00	N/A	N/A
Yes		3,259	84.61		
No		593	15.39		
**Father takes child for walks/park**	4	3,735	100.00	N/A	N/A
Yes		2,999	80.29		
No		736	19.71		
**Tenure**	1	6,726	100.00	N/A	N/A
Owner, mortgaged, shared owner		4,487	66.71		
Rent, rent-free		2,239	33.29		
**Tenure**	2	6,408	100.00	N/A	N/A
Owner, mortgaged, shared owner		4,501	70.24		
Rent, rent-free		1,907	29.76		
**Tenure**	3	6,493	100.00	N/A	N/A
Owner, mortgaged, shared owner		4,594	70.75		
Rent, rent-free		1,899	29.25		
**Tenure**	4	6,353	100.00	N/A	N/A
Owner, mortgaged, shared owner		4,558	71.75		
Rent, rent-free		1,795	28.25		

**Tenure**	5	1,610	100.00	N/A	N/A
Owner, mortgaged, shared owner		711	44.16		
Rent, rent-free		899	55.84		
**Tenure**	6	6,885	100.00	N/A	N/A
Owner, mortgaged, shared owner		4,733	68.74		
Rent, rent-free		2,152	31.26		
**Smoking Mother**	1	6,728	100.00	N/A	N/A
Not Smoking		4,948	73.54		
Smoking		1,780	26.46		
**Smoking Mother**	5	6,636	100.00	N/A	N/A
Not Smoking		5,225	78.74		
Smoking		1,411	21.26		
**Smoking Mother**	6	6,936	100.00	N/A	N/A
Not Smoking		5,587	80.55		
Smoking		1,349	19.45		
**Rooms**	1	6,727	100	5.50	1.46
**Rooms**	2	6,408	100	5.88	1.55
**Rooms**	3	6,495	100	6.06	1.60
**Rooms**	4	6,349	100	6.20	1.64
**Rooms**	6	6,789	100	6.34	1.64
**SES**	1	6,210	100.00	3.14	1.75
Class I		2,184	35.17		
Class II		367	5.91		
Class III		258	4.15		
Class IV		1,206	19.42		
Class V		2,195	35.35		
**SES**	2	3,671	3,671 100.00	3.54	1.63
Class I		906	24.68		
Class II		131	3.57		
Class III		248	6.76		
Class IV		836	22.77		
Class V		1,550	42.22		
**SES**	3	6,065	100	3.16	1.73
Class I		2,080	34.30		
Class II		290	4.78		
Class III		334	5.51		
Class IV		1,275	21.02		
Class V		2,086	34.39		

**SES**	4	6,032	100	3.20	1.72
Class I		2,022	33.52		
Class II		265	4.39		
Class III		384	6.37		
Class IV		1,222	20.26		
Class V		2,139	35.46		
**Mother Reads to child**	3	6,583	100	N/A	N/A
Yes		6,251	94.96		
No		332	5.04		
**Mother Reads to child**	4	6,449	100	N/A	N/A
Yes		5,830	90.40		
No		619	9.60		
**Father Reads to child**	3	3,907	100	N/A	N/A
Yes		3,246	83.08		
No		661	16.92		
**Father Reads to child**	4	3,795	100.00	N/A	N/A
Yes		2,884	75.99		
No		911	24.01		
**Marital Status**	1	6,730	100	N/A	N/A
Married, in relationship		4,288	63.71		
Single, divorced, separated, widowed		2,442	36.29		
**Marital Status**	3	6,582	100.00	N/A	N/A
Married, in relationship		4,495	68.29		
Single, divorced, separated, widowed		2,087	31.71		
**Marital Status**	4	6,454	100	N/A	N/A
Married, in relationship		4,421	68.50		
Single, divorced, separated, widowed		2,033	31.50		
**Marital Status**	5	6,637	100	N/A	N/A
Married, in relationship		4,546	68.49		
Single, divorced, separated, widowed		2,091	31.51		
**Marital Status**	6	6,933	100	N/A	N/A
Married, in relationship		4,625	66.71		
Single, divorced, separated, widowed		2,308	33.29		
**Finance Issues**	1	6,723	100	N/A	N/A
Financially comfortable		6,086	90.53		
Financial issues		637	9.47		
**Finance Issues**	2	6,408	100	N/A	N/A
Financially comfortable		5,835	91.06		

Financial issues		573	8.94		
**Finance Issues**	6	6,793	100	N/A	N/A
Financially comfortable		6,159	90.67		
Financial issues		634	9.33		
**Alcohol Consumption Mother**	1	6,728	100	N/A	N/A
Monthly or less; Never		3,994	59.36		
Weekly or daily		2,734	40.64		
**Alcohol Consumption Mother**	3	6,581	100	N/A	N/A
Monthly or less; Never		3,646	55.40		
Weekly or daily		2,935	44.60		
**Alcohol Consumption Mother**	4	6,448	100	N/A	N/A
Monthly or less; Never		3,389	52.56		
Weekly or daily		3,059	47.44		
**Alcohol Consumption Mother**	5	6,492	100	N/A	N/A
Monthly or less; Never		4,438	68.36		
Weekly or daily		2,054	31.64		
**Alcohol Consumption Mother**	6	6,654	100	N/A	N/A
Monthly or less; Never		4,523	67.97		
Weekly or daily		2,131	32.03		
**Alcohol Consumption Father**	1	3,857	100	N/A	N/A
Monthly or less; Never		1,281	33.21		
Weekly or daily		2,576	66.79		
**Alcohol Consumption Father**	3	3,906	100	N/A	N/A
Monthly or less; Never		1,323	33.87		
Weekly or daily		2,583	66.13		
**Alcohol Consumption Father**	4	3,792	100	N/A	N/A
Monthly or less; Never		1,278	33.70		
Weekly or daily		2,514	66.30		
**Alcohol Consumption Father**	5	3,965	100	N/A	N/A
Monthly or less; Never		2,142	54.02		
Weekly or daily		1,823	45.98		
**Alcohol Consumption Father**	6	4,164	100	N/A	N/A
Monthly or less; Never		2,204	52.93		
Weekly or daily		1,960	47.07		

Note: MCS phenotype data is available as household panel data. The mother's responses (or father's responses if unavailable) for tenure, rooms, SES, financial difficulties and marital status had been matched to the whole family by assigning the same value to all family members in the same household. Therefore, when calculating descriptive statistics, only the mother's responses had been used for the genotyped target sample for tenure, rooms, SES, financial difficulties and marital status. MCS Total = refers to all individuals who provided DNA data (cohort member aged 14 years of age and their biological parents) for which we have phenotype data for

Table S15: Cohort Characteristics for USoc

Environment	Wave	USoc Total			
		Number of participants	% participants	Mean	(SD)
**Age**	N/A	Mixed ages<br>Only individuals over 16 years of age were used for this study (mean age = 52 at wave 1)			
**Gender**	N/A	7,384	100	N/A	N/A
Males		3,103	42.02		
Females		4,281	57.98		
**Income**	1	5,335	100	24.95	14.67
**Income**	2	7,279	100	25.04	14.53
**Income**	3	7,066	100	25.13	14.50
**Income**	4	6,732	100	25.13	14.50
**Income**	5	6,448	100	25.15	14.54
**Income**	6	5,938	100	25.27	14.48
**Income**	7	5,666	100	25.26	14.46
**Income**	8	5,387	100	25.28	14.42
**Income**	9	5,038	100	25.09	14.47
**Rooms**	1	5,359	100	2.93	0.99
**Rooms**	2	7,307	100	2.94	0.99
**Rooms**	3	7.088	100	2.95	.98
**Rooms**	4	6,722	100	2.96	.98
**Rooms**	5	6,442	100	2.97	.98
**Rooms**	6	5,960	100	2.98	0.99
**Rooms**	7	5,719	100	3.00	0.99
**Rooms**	8	5,435	100	2.99	.99
**Rooms**	9	5,055	100	3.01	1.00
**SES**	1	2,963	100	3.31	.89
Class I		100	3.37		
Class II		363	12.25		
Class III		1,198	40.43		
Class IV		1,116	37.66		
Class V		186	6.28		
**SES**	2	4,019	100	3.30	.87
Class I		118	2.94		
Class II		525	13.06		
Class III		1,636	40.71		
Class IV		1,517	37.75		
Class V		223	5.55		

**SES**	3	3,842	100	3.30	.87
Class I		110	2.86		
Class II		497	12.94		
Class III		1,592	41.44		
Class IV		1,429	37.19		
Class V		214	5.57		
**SES**	4	3,640	100	3.30	.87
Class I		106	2.91		
Class II		481	13.21		
Class III		1,485	40.80		
Class IV		1,356	37.25		
Class V		212	5.82		
**SES**	5	3,477	100	3.31	.88
Class I		98	2.82		
Class II		460	13.23		
Class III		1,401	40.29		
Class IV		1,308	37.62		
Class V		210	6.04		
**SES**	6	3,150	100	3.33	.86
Class I		79	2.51		
Class II		401	12.73		
Class III		1,249	39.65		
Class IV		1,245	39.52		
Class V		176	5.59		
**SES**	7	2,975	100	3.34	.86
Class I		73	2.45		
Class II		376<br>12.64	376<br>12.64		
Class III		1,177<br>39.56	1,177<br>39.56		
Class IV		1,171<br>39.36	1,171<br>39.36		
Class V		178<br>5.98	178<br>5.98		
**SES**	8	2,754	100	3.35	.86
Class I		64	2.32		
Class II		344	12.49		
Class III		1,090	39.58		
Class IV		1,084	39.36		
Class V		172	6.25		
**SES**	9	2,485	100	3.35	.87
Class I		59	2.37		

Class II		314	12.64		
Class III		968	38.95		
Class IV		988	39.76		
Class V		156	6.28		
**Financial Issues**	1	5,268	100	N/A	N/A
Financially comfortable		4,753	90.22		
Financial issues		515	9.78		
**Financial Issues**	2	7,258	100	N/A	N/A
Financially comfortable		6,595	90.87		
Financial issues		663	9.13		
**Financial Issues**	3	7,014	0	N/A	N/A
Financially comfortable		6,392	91.13		
Financial issues		622	8.87		
**Financial Issues**	4	6,658	100	N/A	N/A
Financially comfortable		6,108	91.74		
Financial issues		550	8.26		
**Financial Issues**	5	6,387	100	N/A	N/A
Financially comfortable		5,934	92.91		
Financial issues		453	7.09		
**Financial Issues**	6	5,861	100	N/A	N/A
Financially comfortable		5,574	95.10		
Financial issues		287	4.90		
**Financial Issues**	7	5,602	100	N/A	N/A
Financially comfortable		5,341	95.34		
Financial issues		261	4.66		
**Financial Issues**	8	5,346	100	N/A	N/A
Financially comfortable		5,059	94.63		
Financial issues		287	5.37		
**Financial Issues**	9	5,016	100	N/A	N/A
Financially comfortable		4,745	94.60		
Financial issues		271	5.40		
**Tenure**	1	5,331	100	N/A	N/A
Owner, mortgaged, shared owner		4,041	75.80		
Rent, rent-free		1,290	24.20		
**Tenure**	2	7,287	100	N/A	N/A
Owner, mortgaged, shared owner		5,581	76.59		
Rent, rent-free		1,706	23.41		
**Tenure**	3	7,075	100	N/A	N/A

Owner, mortgaged, shared owner		5,445	76.96		
Rent, rent-free		1,630	23.04		
**Tenure**	4	6,703	100	N/A	N/A
Owner, mortgaged, shared owner		5,188	77.40		
Rent, rent-free		1,515	22.60		
**Tenure**	5	6,431	100	N/A	N/A
Owner, mortgaged, shared owner		4,980	77.44		
Rent, rent-free		1,451	22.56		
**Tenure**	6	5,949	100	N/A	N/A
Owner, mortgaged, shared owner		4,651	78.18		
Rent, rent-free		1,298	21.82		
**Tenure**	7	5,703	100	N/A	N/A
Owner, mortgaged, shared owner		4,471	78.40		
Rent, rent-free		1,232	21.60		
**Tenure**	8	5,397	100	N/A	N/A
Owner, mortgaged, shared owner		4,259	78.91		
Rent, rent-free		1,138	21.09		
**Tenure**	9	5,028	100	N/A	N/A
Owner, mortgaged, shared owner		4,033	80.21		
Rent, rent-free		995	19.79		
**Employment**	1	5,306	100	N/A	N/A
employed/retired/maternity leave/apprenticeship		4,491	84.64		
unemployed/education/sick/in care/unpaid/gov training		815	15.36		
**Employment**	2	7,253	100	N/A	N/A
employed/retired/maternity leave/apprenticeship		6,183	85.25		
unemployed/education/sick/in care/unpaid/gov training		1,070	14.75		
**Employment**	3	7,040	100	N/A	N/A
employed/retired/maternity leave/apprenticeship		6,093	86.55		
unemployed/education/sick/in care/unpaid/gov training		947	13.45		
**Employment**	4	6,700	100	N/A	N/A
employed/retired/maternity leave/apprenticeship		5,925	88.43		
unemployed/education/sick/in care/unpaid/gov training		775	11.57		
**Employment**	5	6,419	100	N/A	N/A
employed/retired/maternity leave/apprenticeship		5,713	89.00		

unemployed/education/sick/in care/unpaid/gov training		706	11.00		
**Employment**	6	5,913	100	N/A	N/A
employed/retired/maternity leave/apprenticeship		5,379	90.97		
unemployed/education/sick/in care/unpaid/gov training		534	9.03		
**Employment**	7	5,650	100	N/A	N/A
employed/retired/maternity leave/apprenticeship		5,171	91.52		
unemployed/education/sick/in care/unpaid/gov training		479	8.48		
**Employment**	8	5,366	100	N/A	N/A
employed/retired/maternity leave/apprenticeship		4,930	91.87		
unemployed/education/sick/in care/unpaid/gov training		436	8.13		
**Employment**	9	5,015	100	N/A	N/A
employed/retired/maternity leave/apprenticeship		4,623	92.18		
unemployed/education/sick/in care/unpaid/gov training		392	7.82		

Notes: USoc Total = refers to all individuals who submitted the biomedical survey at the age of 44 and for whom we have phenotype data for

Table S16: Cohort Characteristics for NCDS - childhood

Environment	Age	NCDS Total			
		Number of participants	% Participants	Mean	(SD)
**Gender - children**	N/A	5,288	100	N/A	N/A
Males		2,620	49.55		
Females		2,668	50.45		
**Financial difficulties**	7	4,266	100.00	N/A	N/A
No		4,004	93.86		
Yes		262	6.14		
**Financial difficulties**	11	4,481	100.00	N/A	N/A
No		4,078	91.01		
Yes		403	8.99		
**Financial difficulties**	16	3,987	100.00	N/A	N/A
No		3,654	91.65		
Yes		333	8.35		
**Father's involvement in childcare**	7	4,594	100.00	N/A	N/A
Yes		4,171	90.79		
No		423	9.21		
**Father's involvement in childcare**	11	4,379	100.00	N/A	N/A
Yes		3,970	90.66		
No		409	9.34		
**Father's interest in child's education**	7	3,114	100	N/A	N/A
Yes		2,464	79.13		
No		650	20.87		
**Father's interest in child's education**	11	3,328	100.00	N/A	N/A
Yes		2,672	80.29		
No		656 19.7	19.71		
**Father's interest in child's education**	16	3,109	100.00	N/A	N/A
Yes		2,506	80.60		
No		603	19.40		
**Father takes child on walks/outings**	7	4,585	100.00	N/A	N/A
Yes		4,362	95.14		
No		223	4.86		
**Father takes child on walks/outings**	11	4,412	100.00	N/A	N/A
Yes		4,064	92.11		

No		348	7.89		
**Mother's interest in child's education**	7	4,496	100.00	N/A	N/A
Yes		3,901	86.77		
No		595	13.23		
**Mother's interest in child's education**	11	4,061	100.00	N/A	N/A
Yes		3,520	86.68		
No		541	13.32		
**Mother's interest in child's education**	16	3,448	100.00	N/A	N/A
Yes		2,882	83.58		
No		566	16.42		
**Mother takes child on walks/outings**	7	4,707	100.00	N/A	N/A
Yes		4,658	98.96		
No		49	1.04		
**Mother takes child on walks/outings**	11	4,561	100.00	N/A	N/A
Yes		4,335	95.04		
No		226	4.96		
**Number of rooms child**	7	4,715	100	4.82	1.30
**Number of rooms child**	11	4,608	100	5.00	1.30
**Number of rooms child**	16	4,051	100	4.96	1.48
**SES child**	0	4,881	4,881<br>100.00	2.96	.87
Class I		375	7.68		
Class II		602	12.33		
Class III		2,981	61.07		
Class IV		687	14.07		
Class V		236	4.84		
**SES child**	7	4,601	100.00	3.01	.88
Class I		233	5.06		
Class II		793	17.24		
Class III		2,579	56.05		
Class IV		709	15.41		
Class V		287	6.24		
**SES child**	11	4,349	100.00	3.07	.89
Class I		200	4.60		
Class II		711	16.35		
Class III		2,311	53.14		
Class IV		858	19.73		
Class V		269	6.19		

**SES child**	16	3,722	100.00	3.11	.87
Class I		160	4.30		
Class II		512	13.76		
Class III		2,024	54.38		
Class IV		799	21.47		
Class V		227	6.10		
**Tenure child**	7	4,561	100.00	N/A	N/A
Owns/part-owns		2,008	44.03		
rents		2,553	55.97		
**Tenure child**	11	4,599	100.00	N/A	N/A
Owns/part-owns		2,166	47.10		
rents		2,433	52.90		
**Tenure child**	16	4,056	100.00	N/A	N/A
Owns/part-owns		2,077	51.21		
rents		1,979	48.79		
**Employment father**	7	4,934	100.00	N/A	N/A
Employed		4,851	98.32		
Unemployed		83	1.68		
**Employment father**	11	4,800	100.00	N/A	N/A
Employed		4,671	97.31		
Unemployed		129	2.69		
**Employment father**	16	3,790	100.00	N/A	N/A
Employed		3,650	96.31		
Unemployed		140	3.69		

Notes: NCDS Total = refers to all individuals who submitted the biomedical survey at the age of 44 and for whom we have phenotype data for

Table S17: Cohort Characteristics for NCDS – adulthood

Environment	Age	NCDS Total			
		Number of participants	% participants	Mean	(SD)
**Number of rooms adult**	23	4,483	100	2.69	.91
**Number of rooms adult**	33	4,730	100	4.60	1.55
**Number of rooms adult**	42	5,113	100	3.07	.90
**Number of rooms adult**	46	4,853	100	5.48	1.70
**Number of rooms adult**	50	4,651	100	5.40	1.80
**Number of rooms adult**	55	774	100	4.73	1.90
**SES adult**	23	3,627	100	3.00	.76
Class I		121	3.34		
Class II		586	16.16		
Class III		2,201	60.68		
Class IV		621	17.12		
Class V		98	2.70		
**SES adult**	33	4,414	100	3.21	.88
Class I		149	3.38		
Class II		637	14.43		
Class III		1,988	45.04		
Class IV		1,403	31.79		
Class V		237	5.37		
**SES adult**	42	4,437	100	3.32	.86
Class I		139	3.13		
Class II		503	11.34		
Class III		1,838	41.42		
Class IV		1,718	38.72		
Class V		239	5.39		
**SES adult**	46	4,280	100	3.37	.83
Class I		87	2.03		
Class II		475	11.10		
Class III		1,729	40.40		
Class IV		1,750	40.89		
Class V		239	5.58		
**SES adult**	50	4,075	100	3.39	.84
Class I		90	2.21		
Class II		438	10.75		
Class III		1,581	38.80		
Class IV		1,732	42.50		

Class V		234	5.74		
**SES adult**	55	3,518	100	3.39	.85
Class I		75	2.13		
Class II		394	11.20		
Class III		1,355	38.52		
Class IV		1,472	41.84		
Class V		222	6.31		
**Tenure adult**	23	2,679	100	N/A	N/A
Owns/part-owns		1,465	54.68		
rents		1,214	45.32		
**Tenure adult**	33	4,338	100	N/A	N/A
Owns/part-owns		3,609	83.20		
rents		729	16.80		
**Tenure adult**	42	4,986	100	N/A	N/A
Owns/part-owns		4,270	85.64		
rents		716	14.36		
**Tenure adult**	46	4,826	100	N/A	N/A
Owns/part-owns		4,241	87.88		
rents		585	12.12		
**Tenure adult**	50	4,623	100	N/A	N/A
Owns/part-owns		4,035	87.28		
rents		588	12.72		
**Tenure adult**	55	2,533	100	N/A	N/A
Owns/part-owns		2,031	80.18		
rents		502	19.82		
**Smoking adult**	23	4,606	100	N/A	N/A
Non-smoker		1,403<br>30.46	1,403<br>30.46		
Smoker		3,203<br>69.54	3,203<br>69.54		
**Smoking adult**	42	5,127	100	N/A	N/A
Non-smoker		2,313	45.11		
Smoker		2,814	54.89		
**Smoking adult**	50	4,667	100	N/A	N/A
Non-smoker		2,172	46.54		
Smoker		2,495	53.46		
**Employment adult**	23	1,898	100	N/A	N/A
Employed		1,569	82.67		
Unemployed/disabled/in education		329	17.33		
**Employment adult**	42	5,091	100	N/A	N/A
Employed		4,456	87.53		

Unemployed/disabled/in education		635	12.47		
**Employment adult**	50	4,599	100	N/A	N/A
Employed		4,087	88.87		
Unemployed/disabled/in education		512	11.13		
**Employment adult**	55	4,198	100	N/A	N/A
Employed		3,630	86.47		
Unemployed/disabled/in education		568	13.53		

Notes: NCDS Total = refers to all individuals who submitted the biomedical survey at the age of 44 and for whom we have phenotype data for

References

StataCorp. (2011). *Stata Statistical Software: Release 12*. In College Station, TX: StataCorp LP.
